# Synthesis and Evaluation of a Novel PET Radioligand for Imaging Glutaminyl Cyclase Activity as a Biomarker for Detecting Alzheimer’s Disease

**DOI:** 10.1021/acssensors.4c00313

**Published:** 2024-05-08

**Authors:** William J. Behof, Justin R. Haynes, Clayton A. Whitmore, Yiu-Yin Cheung, Mohammed N. Tantawy, Todd E. Peterson, Printha Wijesinghe, Joanne A. Matsubara, Wellington Pham

**Affiliations:** † Vanderbilt University Institute of Imaging Science, Vanderbilt University Medical Center, Nashville, Tennessee 37232, United States; ‡ Department of Radiology and Radiological Sciences, Vanderbilt University Medical Center, Nashville, Tennessee 37232, United States; § Department of Ophthalmology and Visual Sciences, University of British Columbia, Vancouver, BC V5Z3N9, Canada; ∥ Vanderbilt Brain Institute, Vanderbilt University, Nashville, Tennessee 37232, United States; ⊥ Vanderbilt Memory and Alzheimer’s Center, Vanderbilt University Medical Center, Nashville, Tennessee 37212, United States; # Department of Biomedical Engineering, Vanderbilt University, Nashville, Tennessee 37235, United States; ∇ Vanderbilt Ingram Cancer Center, Nashville, Tennessee 37232, United States; ○ Vanderbilt Institute of Chemical Biology, Vanderbilt University, Nashville, Tennessee 37232, United States; ◆ Vanderbilt Institute of Nanoscale Science and Engineering, Vanderbilt University, Nashville, Tennessee 37235, United States

**Keywords:** Alzheimer’s disease, glutaminyl cyclase, PET imaging, pyroglutamate Abeta, [^18^F]PB0822

## Abstract

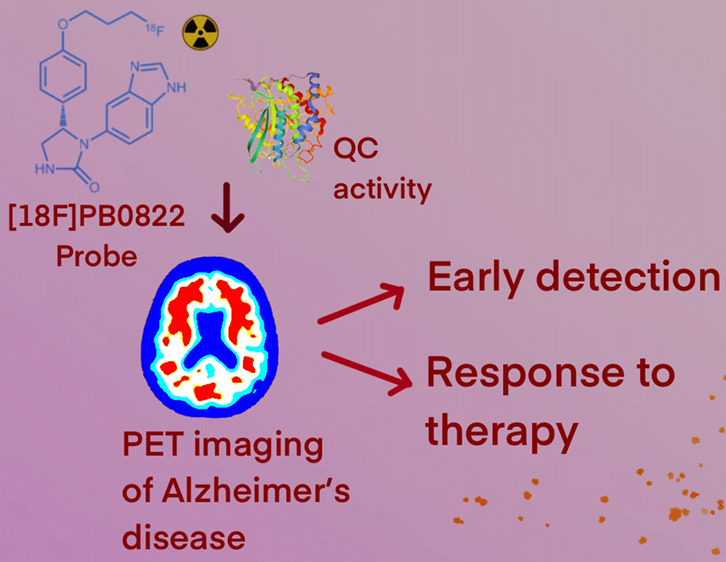

Several new lines of research have demonstrated that a significant number of amyloid-β peptides found in Alzheimer’s disease (AD) are truncated and undergo post-translational modification by glutaminyl cyclase (QC) at the N-terminal. Notably, QC’s products of Abeta-pE3 and Abeta-pE11 have been active targets for investigational drug development. This work describes the design, synthesis, characterization, and in vivo validation of a novel PET radioligand, [^18^F]PB0822, for targeted imaging of QC. We report herein a simplified and robust chemistry for the synthesis of the standard compound, [^19^F]PB0822, and the corresponding [^18^F]PB0822 radioligand. The PET probe was developed with 99.9% radiochemical purity, a molar activity of 965 Ci.mmol^–1^, and an IC_50_ of 56.3 nM, comparable to those of the parent PQ912 inhibitor (62.5 nM). Noninvasive PET imaging showed that the probe is distributed in the brain 5 min after intravenous injection. Further, in vivo PET imaging with [^18^F]PB0822 revealed that AD 5XFAD mice harbor significantly higher QC activity than WT counterparts. The data also suggested that QC activity is found across different brain regions of the tested animals.

Alzheimer’s disease (AD) is the most prevalent cause of dementia among elderly people with unknown etiology.^1^ The cytopathologic hallmarks of AD are the extracellular amyloid-β protein (Abeta) and intracellular neurofibrillary tangles, which lead ultimately to profound neuronal toxicity and tissue atrophy.^2^ Particularly, the Abeta proteins with full-length amino acid residues 1–40 and 1–42 have been the center of research focus for several decades. However, there are other isoforms of Abeta proteins, including the N- and C-terminal truncated, as well as modified analogues. When N-terminal truncation exposes a glutamic acid residue, the amino terminus of Abeta can become cyclized into a five-membered ring, which is a very stable entity.^3^ This post-translational modification is catalyzed by glutaminyl cyclase (QC) to form pyroglutamate Abeta (Abeta-pE).^4^ Two Abeta-pE products of QCs found in AD brains are Abeta-pE with cyclization of the glutamate residues 3 (Abeta-pE3) and 11 (Abeta-pE11), and these have become the topic of considerable study.^3^ The QC-mediated formation of pyroglutamate leads to a more stable protein with a significant loss of electronic charges, thus enhancing the hydrophobicity of the final substrate (Figure 1). It is believed that this hydrophobicity promotes rapid conformational change and protein misfolding into the β-sheet structures and toxic aggregation.^5−7^ Notably, Abeta-pE3 is found as a major species deposited in the plaques and vessels of AD and Down syndrome patients.^8−11^ Abeta-pE3 is also deposited in the brains of several preclinical animal models, albeit at the later stages compared to human cases.^12^ Additionally, this post-translational modification of Abeta is more neurotoxic than other Abeta counterparts.^12,13^

**Figure 1:**
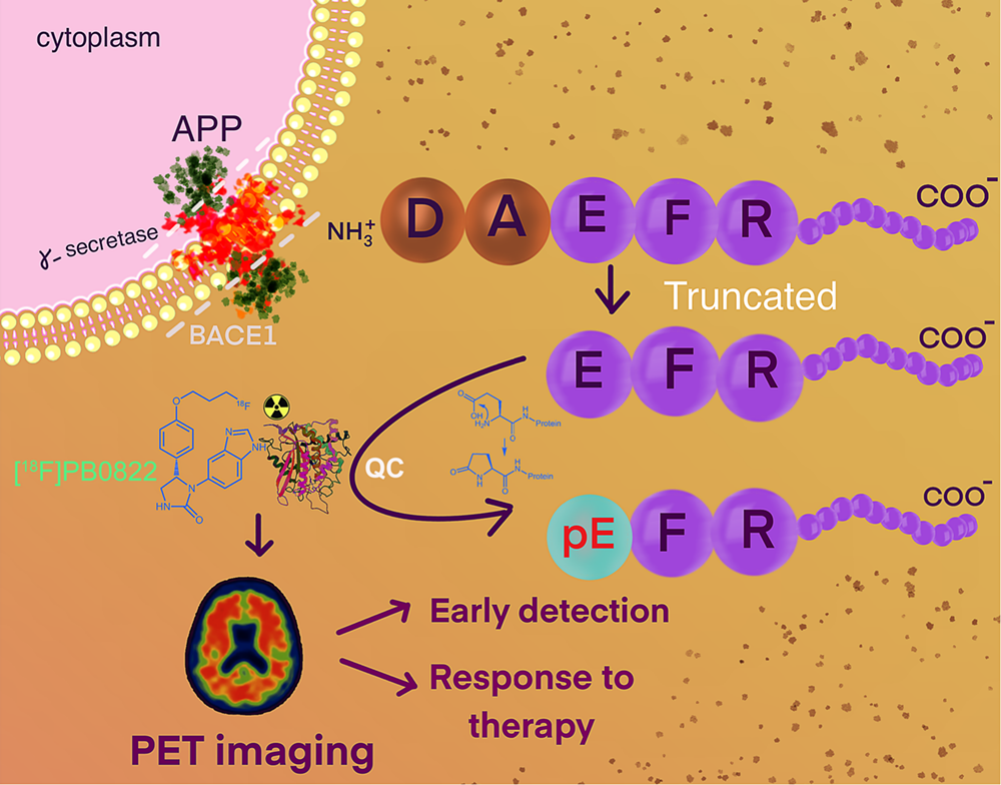
Mechanism of formation of Abeta-pE3 from a truncated Abeta peptide via QC activity. Due to the loss of charged moieties, the product Abeta-pE3 is more hydrophobic and has an increased aggregation propensity. The unique implications of QC in AD represent an ideal target for imaging intervention.

QC belongs to the family of metal-dependent aminoacyltransferases, which is responsible for the conversion of glutaminyl residues at the N-terminus of peptides into pyroglutaminyl peptides.^14^ With an average molecular weight ranging between 33 and 40KDa,^15^ QC is widely distributed in the hippocampus and cortex of mammalian brains.^16,17^ Glutaminyl cyclase mRNA and protein were upregulated in AD patients compared to normal aging individuals and correlated with the existence of larger concentrations of Abeta-pE3 compared to healthy controls.^18,19^ Treatments of different transgenic mouse models of AD with oral doses of a QC inhibitor resulted in reduced Abeta-pE3 burden, followed by diminished plaque formation and improved cognition.^18^ The efficacy and safety of a QC inhibitor, such as varoglutamstat (PQ912), have been reported as promising in clinical trials.^20,21^ Collectively, these data suggest the critical role of QC in the neuropathology of AD. We postulate that if an imaging probe of QC is available, it would help to assess QC activity in vivo, both in preclinical and clinical settings. This probe would serve not only for AD detection but also for staging and imaging response to clinical therapy (Figure 1). Toward that approach, we report herein the development and validation of a novel QC PET radioligand, [^18^F]PB0822, based on the chemical backbone of PQ912.^22^ The tosylate precursor can be obtained in a four-step synthesis. After chiral purification, the enantiomers were converted to the respective [^19^F] analogue and compared to previously reported literature^22^ and commercial sources to identify the S-configuration. The data show no significant change in the IC_50_ value of the [^18^F]PB0822 PET radioligand compared to that of PQ912. Radioisotope labeling was achieved in merely 10 min with 99.9% radiochemical purity and a molar activity of 965 Ci.mmol^–1^. Dynamic PET scans showed that the [^18^F]PB0822 PET radioligand has acceptable pharmacokinetics for in vivo applications in the brain after intravenous injection. The [^18^F]PB0822 probe can specifically detect higher QC activity in the brains of the 5XFAD mouse model than in their WT counterparts.

## Experimental Section

All of the information regarding reagents, chemicals, synthesis, purifications, chiral separation, and characterization of the intermediates, precursor, and standard compound, along with associated instruments, can be found in the Supporting Information (SI).

### Synthesis and Characterization of the Precursor

The synthetic information along with ^1^H NMR, ^13^C NMR, and high-resolution mass spectrometry confirming the described products is included in the SI.

### Chiral Separation

The enantioseparations were performed using the Berger Multigram II HPLC/SFC preparative chromatography (Berger Instruments Inc.) incorporated with the (R,R)-Whelk-O1 column (Regis Technologies, Inc.) with dimensions of 2.1 I.D. × 25 cm length and particle size of 5 μm. The purification was performed with column ambient temperature at 40 °C using a static mobile phase of CO_2_/MeOH/TEA (55/45/0.05, v/v/v) with a flow rate of 20 mL/min. The products were monitored at 214 nm and dried via rotary evaporation.

#### [^18^F]PB0822 Radiotracer Synthesis

The tosylate precursor was labeled with [^18^F]fluoride with an optimized condition, using [^18^F]KF and K_2.2.2_ in acetonitrile at 100 °C for 10 min with the unprotected benzimidazole. The radiochemical purity and the identity of the [^18^F]PB0822 PET radioligand were characterized by an analytical HPLC system, equipped with a UV absorption detector (λ = 254 nm) and a radioisotope detector (Bioscan Flow-Count). The HPLC setup comprises a Phenomenex Luna 5 μm C18(2) (00G-4252-E0, 100 Å, 250 × 4.6 mm) column with a typical mobile phase of acetonitrile and ammonium formate (30:70% of 0.1 M, pH = 6.5) at a flow rate of 1 mL/min. The identity of [^18^F]PB0822 was confirmed by comparing the retention time with the coinjected and standard compound [^19^F]PB0822 (RT = 10.56 min) along with the gamma peak (RT = 10.88 min). [^18^F]PB0822 was obtained with a 1.2% yield (nondecay corrected) at EOS with 99.9% radiochemical purity and with a molar activity of the radioligand at 965 Ci.mmol^–1^.

#### Animals

A colony of 5XFAD mice obtained from Jackson Laboratories was maintained by crossing with WT C57BL/6J as we reported in the past.^23^ The animals were genotyped by a polymerase chain reaction (PCR) using DNA obtained from tail or ear tissue samples. After PCR amplification, DNA product was analyzed using a 1% agarose gel, amyloid precursor protein (APP) transgene = 377 bp and presenilin 1 (PSEN1) transgene = 608 bp. 5XFAD mice were maintained as heterozygous. Animal experiments were conducted in accordance with the guidelines established by the Vanderbilt University’s Institutional Animal Care and Use Committee (IACUC) and the Division of Animal Care and approved by Vanderbilt IACUC, protocol number M1700044.

#### Dynamic PET Imaging

The dynamic acquisition was divided into twelve 5 s frames, four 60 s frames, five 120 s frames, three 5 min frames, and four 10 min scans. The data from all possible lines of response (LOR) were saved in the list mode raw data format. The raw data were then binned into 3D sinograms with a span of 3 and a ring difference of 79. The images were reconstructed into transaxial slices (128 × 128 × 159) with voxel sizes of 0.03882 × 0.03882 × 0.0796 cm^3^ using the OSEM3D/MAP algorithm with 2 OSEM3D iteration, followed by MAP 16 subsets, 18 iterations, beta of 1.47097, and MAP resolution of 1.5 mm. For anatomical coregistration, immediately following the PET scans, the mice received a CT scan in a NanoSPECT/CT (Mediso, Washington, DC. at an X-ray beam intensity of 90 mAs and an X-ray peak voltage of 45 kVp. The CT images were reconstructed into 170 × 170 × 300 voxels at a voxel size of 0.4 × 0.4 × 0.4 mm^3^. The PET/CT images were uploaded into Amide software (www.sourceforge.com) and coregistered to each other based on bed position and to an MRI template made in-house. Volumetric regions-of-interest (ROIs) were drawn around the cortex, hippocampus, striatum, thalamus, and cerebellum in addition to the whole brain in the template and superimposed onto the PET images. The PET images were normalized to the injected dose and animal weight, and the time-activity curves (TACs) of the mean activity within the ROIs were estimated for the entire duration of the scans in SUV (standard uptake values).

#### Glutaminyl Cyclase Inhibition Assays

This assay was developed based on the prior report with some modifications.^24^ Human recombinant glutaminyl peptide cyclotransferase (QC) and human recombinant pyroglutamyl peptidase I (PGPEP1) were obtained from ProSpec-Tany TechnoGene Ltd. (Rehovot, Israel). PQ912 was purchased from Aobious Inc. (Gloucester, MA). PB-0822 was developed in-house, as described elsewhere in this paper.

Assays were performed in 96-well plates at 37 °C in a buffer solution consisting of pH 6.0 HEPES with 1 mM dithiothreitol (DTT) and 20% (v/v) glycerol. H-Gln-AMC hydrobromide salt (Bachem Americas Inc., Torrance, CA) was used as the substrate. Each assay replicate contained 0.125 μg of QC and 1.25 μg of PGPEP1. After a 15 min preincubation period for all reagents at 37 °C, reactions were initiated by the addition of QC to a solution containing PGPEP1, 80 μM H-Gln-AMC, and varying concentrations (0–1000 nM) of PQ912 or either isomer of PB0822. Immediately after the addition of QC, fluorescence emission at 460 nm (380 nm_ex_) was measured every minute for 2 h using a microplate reader (Biotek Industries, Agilent Technologies, Winooski, VT, USA). Data were exported to Excel, and fluorescence values were converted to the product formation rate by using the equation generated from a standard curve made with 7-amino-4-methylcoumarin (Sigma-Aldrich Inc., St. Louis, MO) under assay conditions. IC_50_ values were calculated using an online calculator from AAT Bioquest (https://www.aatbio.com/tools/ic50-calculator).

### Cardiac Perfusion Procedure and Tissue Collection

All of the IHC brain data were collected on perfused mice. Basically, deeply anesthetized mice were laid on a stainless-steel tray half filled with crushed ice, and the thoracic cavity was opened with a scalpel after making 5–6 cm midline incision starting from the abdominal area. After careful separation of the liver from the diaphragm, the thoracic opening was held open with the assistance of a retractor. Perfusion was commenced as described in the past^23,25,26^ by slowly injecting the left ventricle with ice-cold PBS (1×) buffer pH 7.4 (30 mL) toward the ascending aorta using a 25 G syringe, while the right atrium was quickly snipped off using a curved-point squeeze-snip scissor to facilitate drainage of the systemic venous return. After perfusion with PBS, the process was repeated with 4% paraformaldehyde (PFA, pH 7.4, 30 mL). When completed, brain and other tissues were harvested for preservation as described before.^27,28^

#### Immunohistochemistry

Brains collected from paraformaldehyde perfused mice were embedded in an OCT and cut into coronal sections (8–10 μm) using a Tissue-Tek cryostat and mounted onto charged glass slides. Prior to staining, slides were washed with PBS (10 min); then, they were treated with blocking buffer (5% normal goat serum, 0.2% Triton X-100, 0.5% bovine albumin in PBS) for 1h at room temperature. The treated sections were then incubated overnight at 4 °C with primary rabbit antipyroglutamate antibody (1:500 dilution, Novus Biologicals, Littleton, CO, USA, catalog number: NBP1-44048). Slides were washed with PBS (3×) for 10 min each, and the sections were subsequently incubated with secondary antibody goat antirabbit Alexa Fluor 647 (1:500 dilution, Thermo Fisher Scientific, Carlsbad, CA, USA, catalog number: A-21245) for 30 min at room temperature. The sections were then washed with PBS twice for 10 min and once for 30 min and coverslipped with an antifade mounting medium (Vector Laboratories, Burlingame, CA, catalog number: H-1200-10) before observation under a fluorescence microscope.

### Quantitative Data Analysis

Quantification of PET imaging and IHC signals was performed using imageJ software. Manual regions-of-interest (ROIs) were drawn and thresholded using identical parameters across samples before counting the pixel intensity. Then, the data were imported to GraphPad Prism version 10 for Mac (Graphpad Software, San Diego, CA, USA) for statistical analysis. Significant differences between two independent groups were determined and compared using a paired parametric *t*-test. Significance is reported when the probability value <0.05.

## Results and Discussion

### Design of a PET Precursor for Radioisotope Labeling

The chemical development of imaging agents offers many labeling choices, but what sets PET chemistry apart most may be the ability to maintain identical structures or ones with very small changes from the targeted ligands. Further, the sensitivity of PET imaging has great implications in neuroimaging. Not only has it enabled the use of radioligands with low doses, at subpharmacological levels, but it also accommodates probes that have low-to-moderate bioavailability in the brain, which would be otherwise impossible to realize with other imaging modalities.

**Figure 2:**
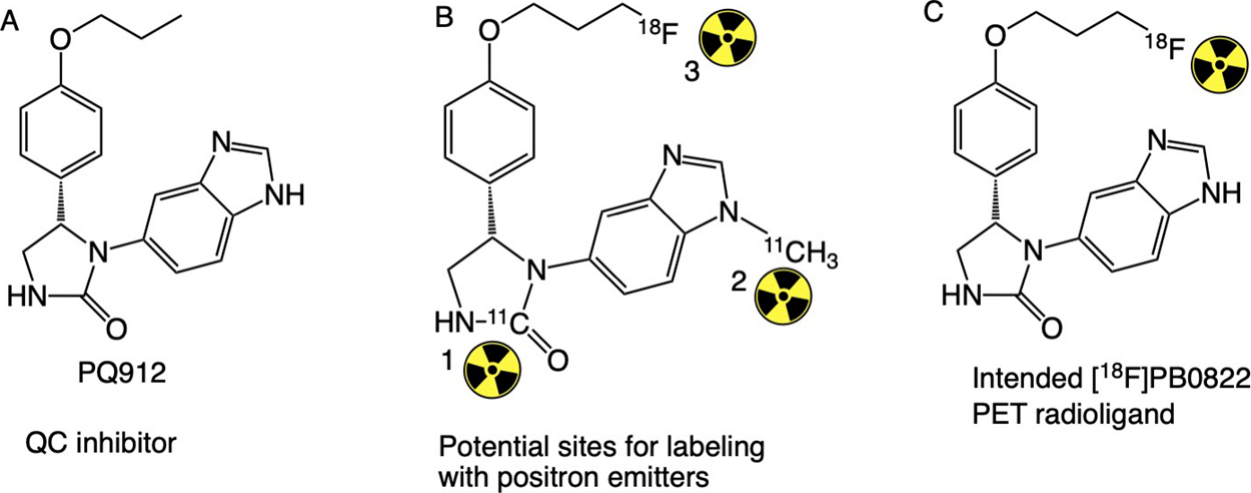
Radioisotope labeling strategy. (A) QC inhibitor PQ912; (B) potential sites on PQ912 where a positron emitter can be labeled with either [^11^C] or [^18^F] radioisotope; and (C) chemical structure of the [^18^F]PB0822 PET radioligand as a modification from PQ912.

Three potential positions exist to mimic PQ912 as a positron emitter (Figure 2). In a typical experiment, [^11^C]CO_2_ can serve as a synthon for [^11^C]carboxylation, which can be used as an intermediate for direct [^11^C] labeling. Several methods have been developed and reported in the past, including the successful synthesis of [^11^C]PQ912.^29,30^ The [^11^C]CO_2_ fixation chemistry was also reported for the synthesis of [^11^C]urea of another QC PET radioligand called [^11^C]QZ.^31^ The advantage of direct [^11^C] labeling is, obviously, to maintain the identical structure of the parent QC inhibitors. Another labeling route comes from the [^11^C]methylation of the amino group on the imidazole ring. The strength of direct [^11^C] labeling, however, is also its shortcoming. As much as chemists may appreciate the convenience of the intrinsic incorporation of the radioisotopes and retaining the same biological activity, the short half-life of the [^11^C]carbon impedes robust in vivo application. Further, the short half-life of [^11^C]-probes may be impractical due to the compensatory high radioactivity exposure to chemists, as well as testing subjects. Thus, we opted to develop a [^18^F]probe in this work to overcome these issues, as well as improve logistical support, where a tosylate precursor was generated for aliphatic [^18^F]fluoride radiolabeling.

### Synthesis of the Precursor for [^18^F]Fluoride Labeling and the [^19^F] Standard Compound

**Figure 3:**
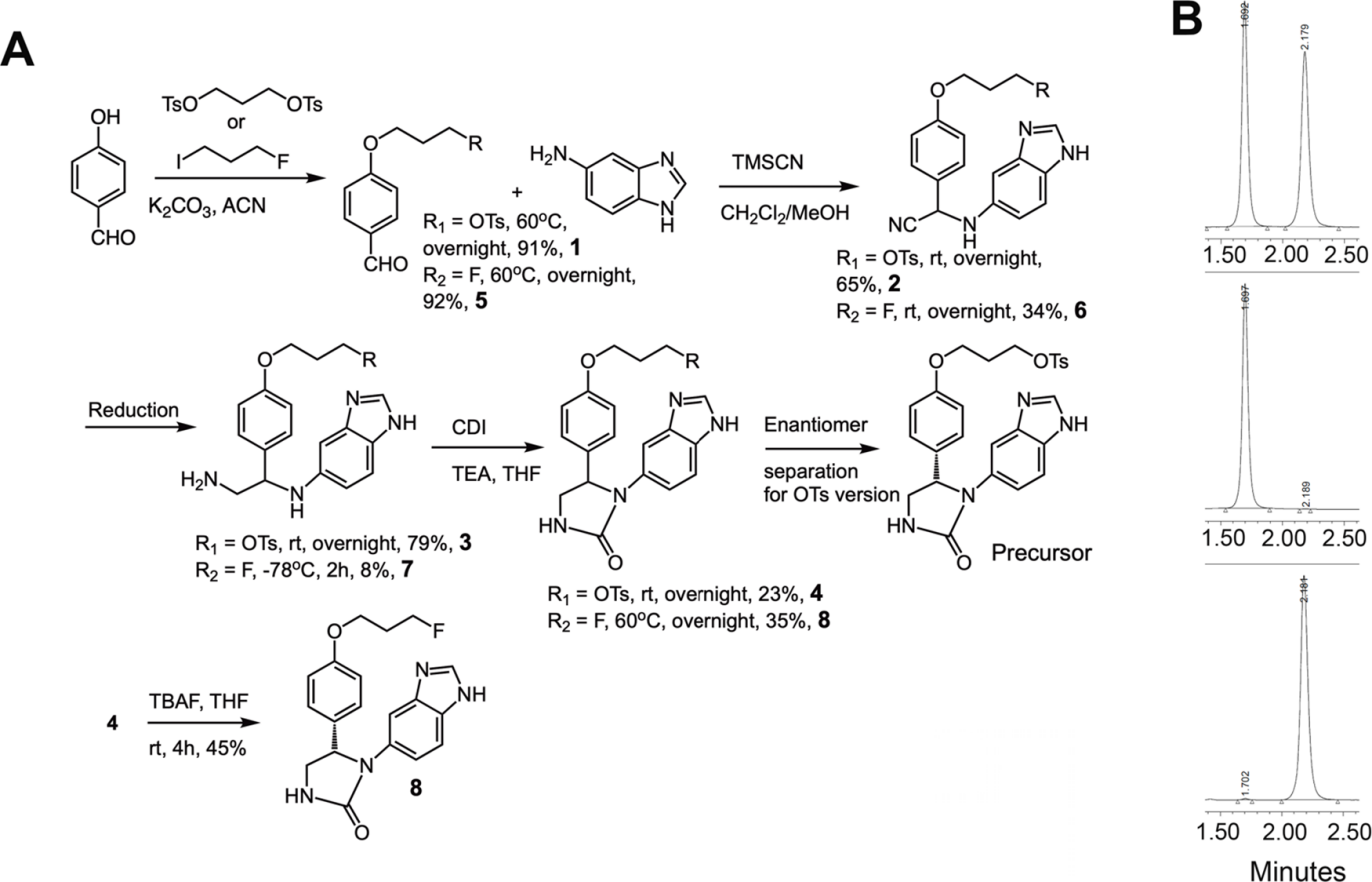
Synthetic chemistry and characterization. (A) Optimized scheme of the synthesis of the tosylate precursor for [^18^F]F^–^ labeling and the standard [^19^F]PB0822; (B) HPLC profiles for chiral separation of the tosylate precursor: (top) racemic compound; (middle) S-enantiomer; (bottom) R-enantiomer.

In this optimized and reproducible scheme of synthesis (Figure 3A), the reaction started with making the tosylate compound **1** (Figures S1–S2, SI). A Strecker reaction was utilized in the next step between the aldehyde and the aminated benzimidazole in the presence of trimethysilyl cyanide (TMSCN) to afford the cyanomethylated amine **2** (Figures S3–S4, SI). An overnight hydrogenation reaction enabled the reduction of nitrile into an amine **3** catalyzed by Pd/C at 120 psi (Figures S5–S6, SI). Finally, successful ring closure was achieved by treating aminated **3** with 1,1′-carbonyldiimidazole (CDI) to provide the desired precursor **4** with modest yield (Figures S7–S8, SI). The enantiomers of the precursor were separated using a chiral column (Figure 3B and additional information is available in SI, Figure S9), upon conversion to the [^19^F] versions, each enantiomer was subjected to the bioassay described in Figure 5; only the S-configuration was recognized by the QC, henceforth only the S-configuration isomer is discussed hereafter.

The chemical design of this PET precursor is unique for two reasons. First, we decided to leave the amine on benzimidazole unprotected. During the course of this work, we found that Boc-protected benzamidine is very labile; it can easily be removed with a trace of TFA present in the HPLC buffer. The active species emanated during Boc deprotection contributes to further destabilizing the compound. Second, the tosylate group was incorporated in the early synthetic steps. Initially, we were uncertain whether it would survive in the Strecker synthesis or during the hydrogenation. Particularly, deoxygenation of aryl tosylates under a palladium catalyst has been reported in the past.^32^ To our delight, the tosylate group survived both reactions. Aside from being the ideal leaving group for [^18^F]F^–^ labeling, incorporating tosylates right from the beginning of the scheme has two goals: one is to serve as a protecting group for the hydroxyl moiety; second, tosylates have the propensity for forming crystals even with a few milligrams, offering an impeccable opportunity for characterization of the chiral products.

In a conventional approach to test the robustness of fluorine labeling with tosylate as a leaving group, particularly in the presence of free amines, precursor **4** was treated with tetra-*n*-butylammonium fluoride (TBAF) at room temperature to provide product **8** with reasonable yield (detailed synthesis is in SI). However, large-scale synthesis of [^19^F]PB0822 for use as a standard analogue was achieved using identical chemical steps when obtaining the tosylate precursor **4**, except 1-fluoro-3-iodopropane was used in the first step of the reaction instead of 1,3 bis-tosylate propane (Figure 3A) (detailed synthesis can be found in SI, Figures S10–S17).

### [^18^F]PB0822 Synthesis and Characterization

**Figure 4:**
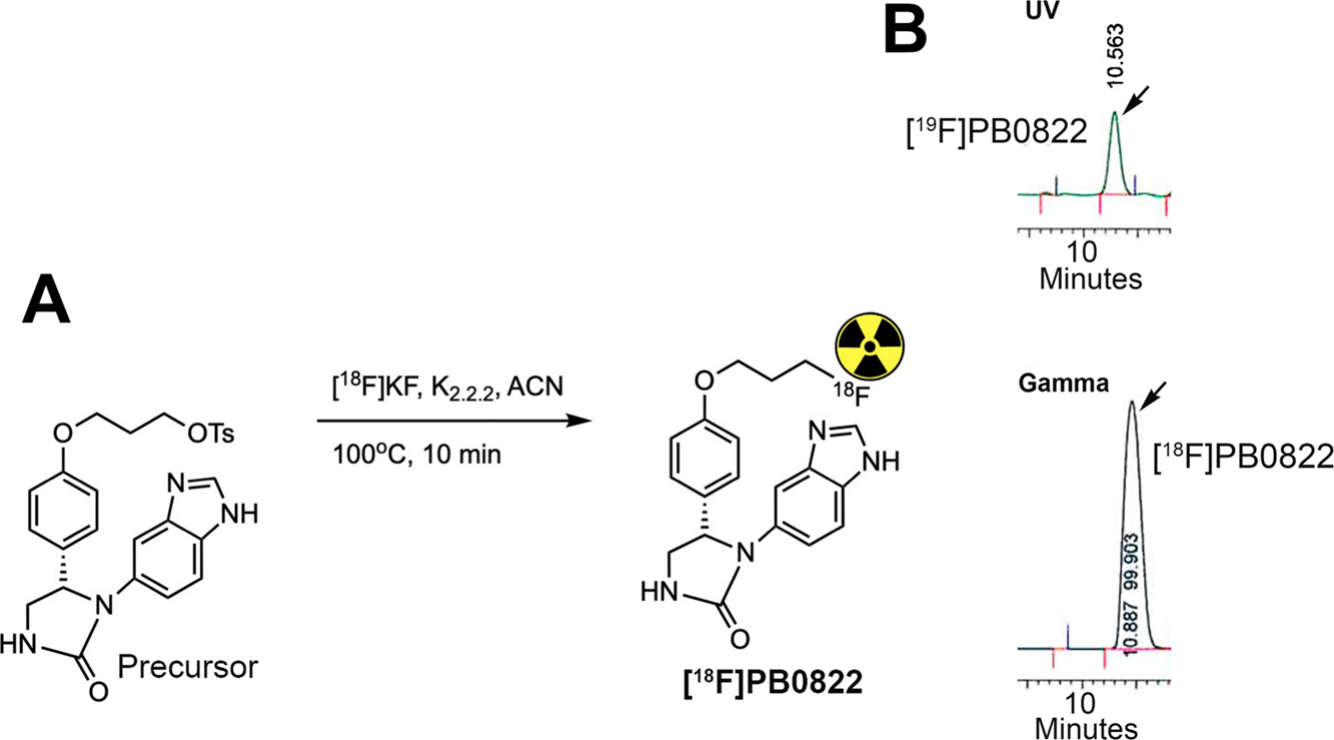
Radioisotope labeling. (A) [^18^F]F^–^ labeling of the nonprotected precursor using a conventional method; (B) HPLC chromatograms of the standard [^19^F]PB0822 coinjected with the [^18^F]PB0822 PET radioligand, confirmed by UV and gamma detector, respectively.

As anticipated, we successfully labeled the tosylate precursor with the [^18^F]fluoride radioisotope using a conventional reaction method in the presence of an unprotected benzimidazole (Figure 4A). Both the precursor and the labeled product are stable at elevated temperatures. After optimizing the reaction conditions, i.e., using acetonitrile as a solvent at 100 °C for 10 min, identical batches (*n* = 5) of synthesis were performed to confirm reproducibility. The identity and purity of the labeled product [^18^F]PB0822 were confirmed using an analytical HPLC. The retention time detected by the gamma detector for [^18^F]PB0822 was confirmed by comparing it with that of the standard compound (Figure 4B) with 99.9% radiochemical purity and a molar activity of the radioligand at 965 Ci.mmol^–1^. Full characterization data, and HPLC conditions/parameters, including the mobile phase, can be found in Figure S18, SI.

#### [^19^F]PB0822 Standard Compound Retains Comparable IC_50_ Value as of PQ912

**Figure 5:**
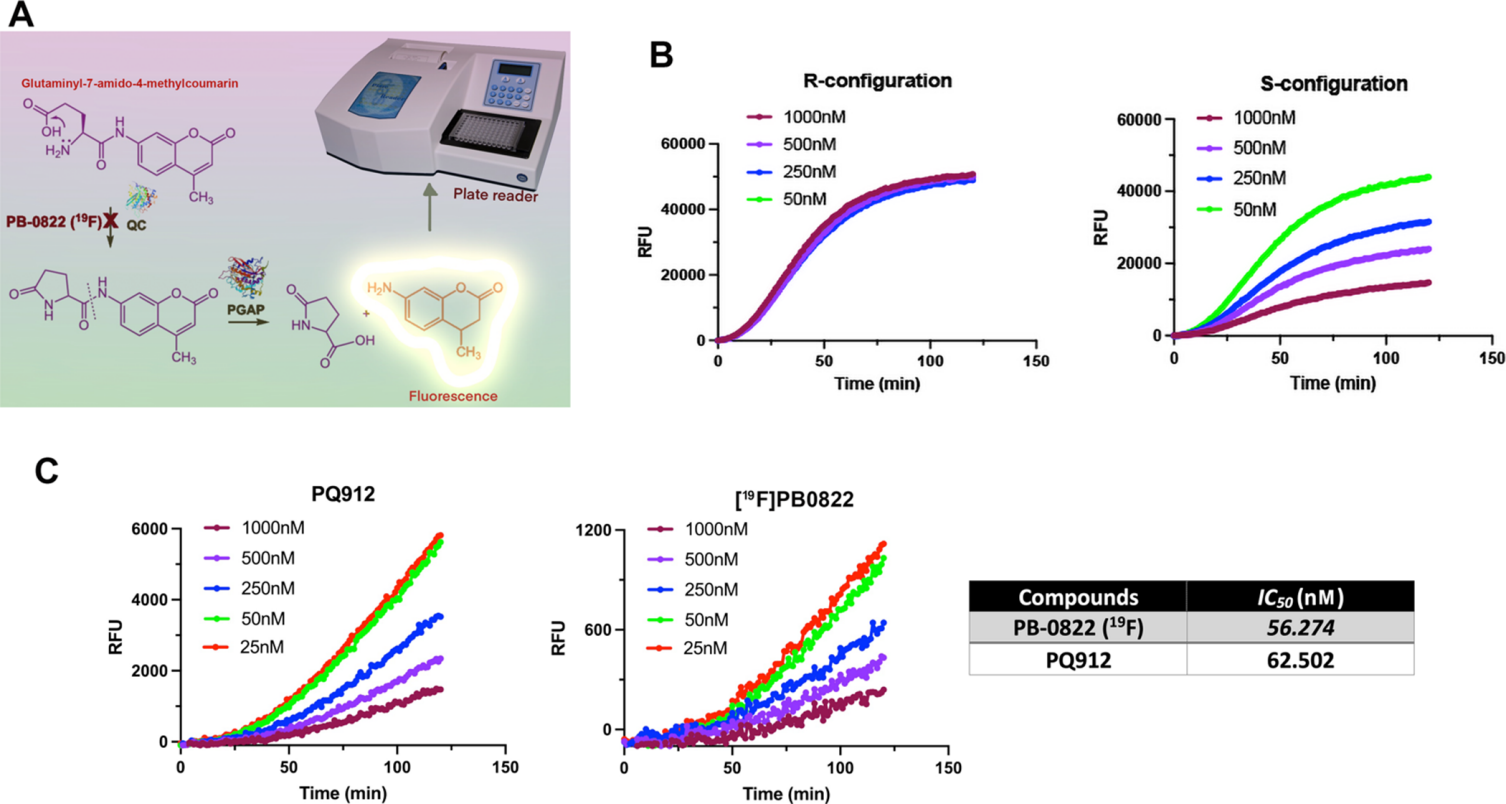
Characterization of [^19^F]PB0822 for QC binding specificity. (A) Schematic description of the assay to characterize the QC inhibitory effect of PB-0822 using glutaminyl-7-amido-4-methylcoumarin as a conditioned signal readout substrate; (B) among the tested enantiomers, only S-[^19^F]PB0822 is recognized by QC and resulted in the attenuation of the fluorescent signal; (C) assay revealed that IC_50_ of S-[^19^F]PB0822 is comparable to that of PQ912.

We used a reported fluorescence assay^24^ with some modifications to assess the specificity among the isolated enantiomers of [^19^F]PB0822 for QC. The overall idea about this assay is depicted in Figure 5A. As the amino group of coumarin dye (AMC) is incorporated into glutamic acid, perturbation of the electronic propagation of coumarin results in quenching of the fluorescence signal. In the presence of QC, pyroglutamate-AMC is formed through cyclization of the N-terminal glutamate and the carboxylic side chain. Then, pyroglutamyl peptidase 1 (PGPEP1), an enzyme specific for pyroglutamyl, cleaves the substrate at the amide bond and releases the aminated coumarin, which restores the fluorescence. In the presence of a specific QC inhibitor, QC activity will be hindered, resulting in a reduced fluorescence output. The data showed that only [^19^F]PB0822 with S-configuration can attenuate the fluorescence in the assay, suggesting its targeted specificity for the QC enzyme (Figure 5B). Furthermore, the probe inhibited QC activity at low-end nanomolar concentrations and in a dose-dependent fashion. From this assay, we found that S-[^18^F]PB0822 has a comparable IC_50_ value (56.274 nM) compared to PQ912 (62.502 nM). In contrast, the assay confirmed that QC did not recognize the R-conformation, despite testing under identical reaction conditions and concentrations as described for the S-counterpart.

Fitting the tested concentrations of PQ912 and those of [^19^F]PB0822 in the regression model resulted in comparable IC_50_ values for both compounds (Figure 5C). The data suggested that adding a fluorine atom to PQ912 does not alter QC recognition, binding specificity, and potency.

#### [^18^F]PB0822 PET Radioligand Detects QC in the Brains of 5XFAD Mice

**Figure 6:**
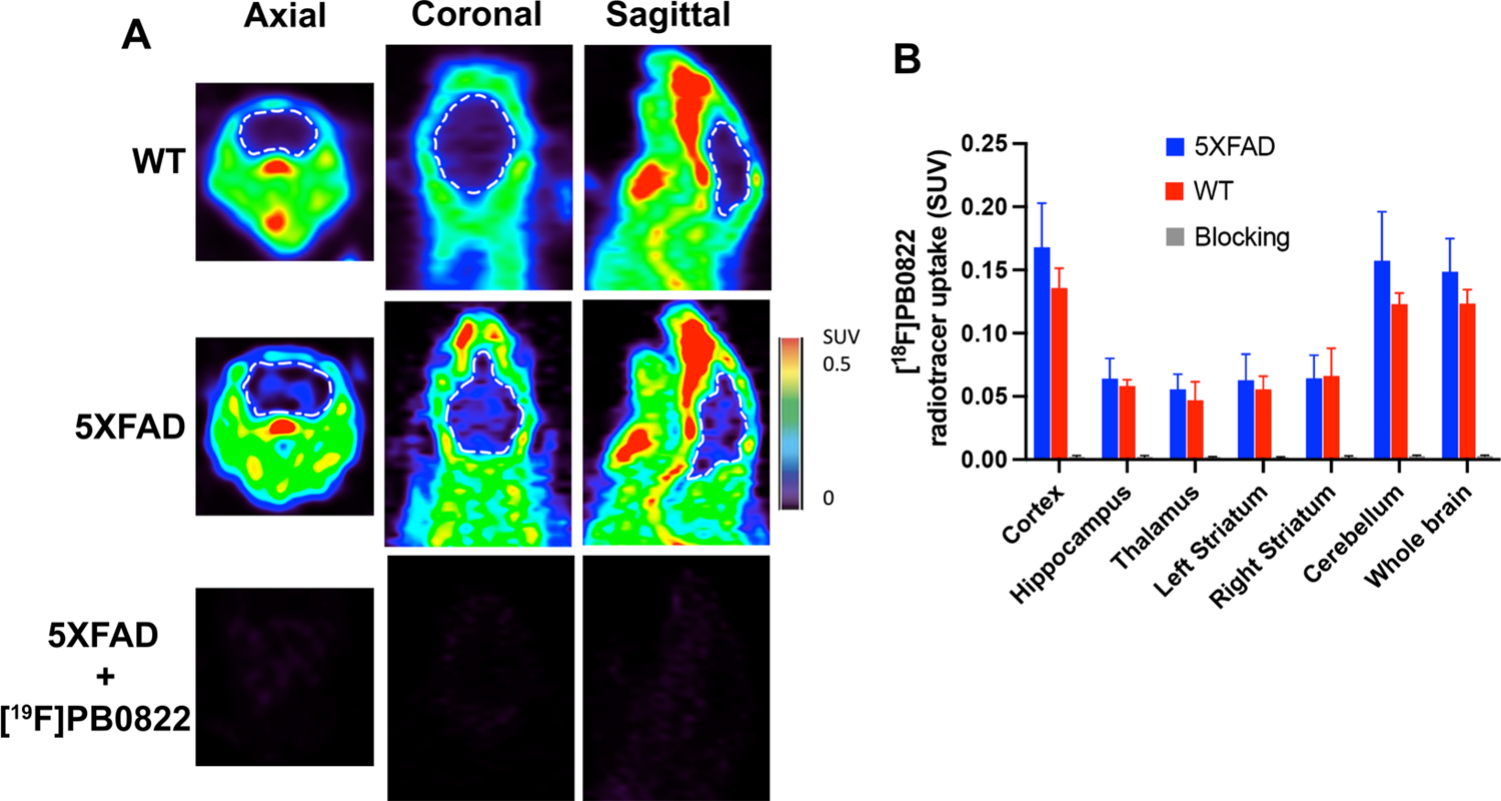
In vivo PET imaging of QC activity in the brains with [^18^F]PB0822. (A) Representative axial, coronal, and sagittal view of WT (*n* = 5) and 5XFAD (*n* = 11) brains (highlighted by discontinued white dots) after animals were injected with the [^18^F]PB0822 radioligand 30 min before 20 min PET scans. In a blocking study (bottom row), selected 5XFAD mice (*n* = 3) were injected with standard [^19^F]PB0822 (compound **8**) minutes prior to injecting the [^18^F]PB0822 probe, resulting in a loss of signal; (B) the detected PET signal representing specific uptake in brain regions was quantified and presented as SUV; *p* = 0.02 between WT vs 5XFAD; *p* = 0.002 between 5XFAD vs blocking group.

With the availability of a novel QC PET radioligand, we demonstrated for the first time noninvasive PET imaging data for the visualization of QC activity in the brain. [^18^F]PB0822 has a cLog*P* value of 1.54, which is a good predictive index for BBB penetration. It has been shown in the past that another QC PET radioligand, [^11^C]PBD150, could not permeate the BBB with the cLog*P* value less than 1.0.^33^ In this triple-blinded study, animals’ IDs and types were not revealed to the team that performed tail vein injection and imaging of animals as well as to the imaging analysis team. The WT (n = 5) and 5XFAD (*n* = 11) mice received equivalent intravenous injection doses (400 μCi/0.1–0.2 mL) via the tail veins and were imaged immediately for a 75 min dynamic scan or imaged 30 min after treatment for a 20 min scan. In the 30 min postinjection cohorts, a higher PET signal was detected in 5XFAD brains as compared to WT brains, suggesting increased QC activity in an AD mouse model (Figure 6A). Quantitative analysis of the SUV data showed that the PET signal in 5XFAD brains was statistically higher than that of WT counterparts, in most of the brain regions (*p* = 0.02) (Figure 6B). To demonstrate the specificity of the probe, selected 5XFAD mice (*n* = 3) were injected with the “cold” compound of [^19^F]PB0822 (22.5 mM) 5 min prior to injection of the probe. After 30 min of uptake, animals were scanned, and the imaging data showed that the excess amount of the “cold” compound competed with the probe, leading to a near abolishment of the PET signal in the brain (*p* = 0.002) (Figure 6A,B). Similar to the in vivo PET imaging data obtained from other QC radioligand, [^11^C]QZ;^31^ aside from signals in the brain, significant signals were detected in the peripheral area. This observation corroborates with the prior report showing an increased QC activity in the blood of AD subjects.^34^ Taken altogether, the data suggest that [^18^F]PB0822 reported the differential QC activity in the brains of normal versus pathological brains. The in vivo PET imaging in this blind study corroborates with human data reported earlier that QC is widely distributed in AD patients’ cortex^13,18^ and hippocampus.^12,35^ This unique form of Abeta is a major constituent of Abeta deposits in sporadic and familial AD.^10,36,37^ The upregulated QC activity in AD patients correlated with the existence of large concentration of Abeta-pE3.^18^ Our PET imaging data show that 5XFAD mouse brains have higher levels of QC than those of their WT counterparts. Particularly, we observed more QC activity in the cortex and cerebellum compared to that in the hippocampus. Furthermore, our immunohistochemistry data corroborate with PET imaging data as it also showed higher levels of Abeta-pE3 in the cortex than in the hippocampus (Figure 8). Our observation is consistent with reported data, indicating significantly enhanced QC activity in the AD frontal cortex compared to neurological controls.^13^

**Figure 7:**
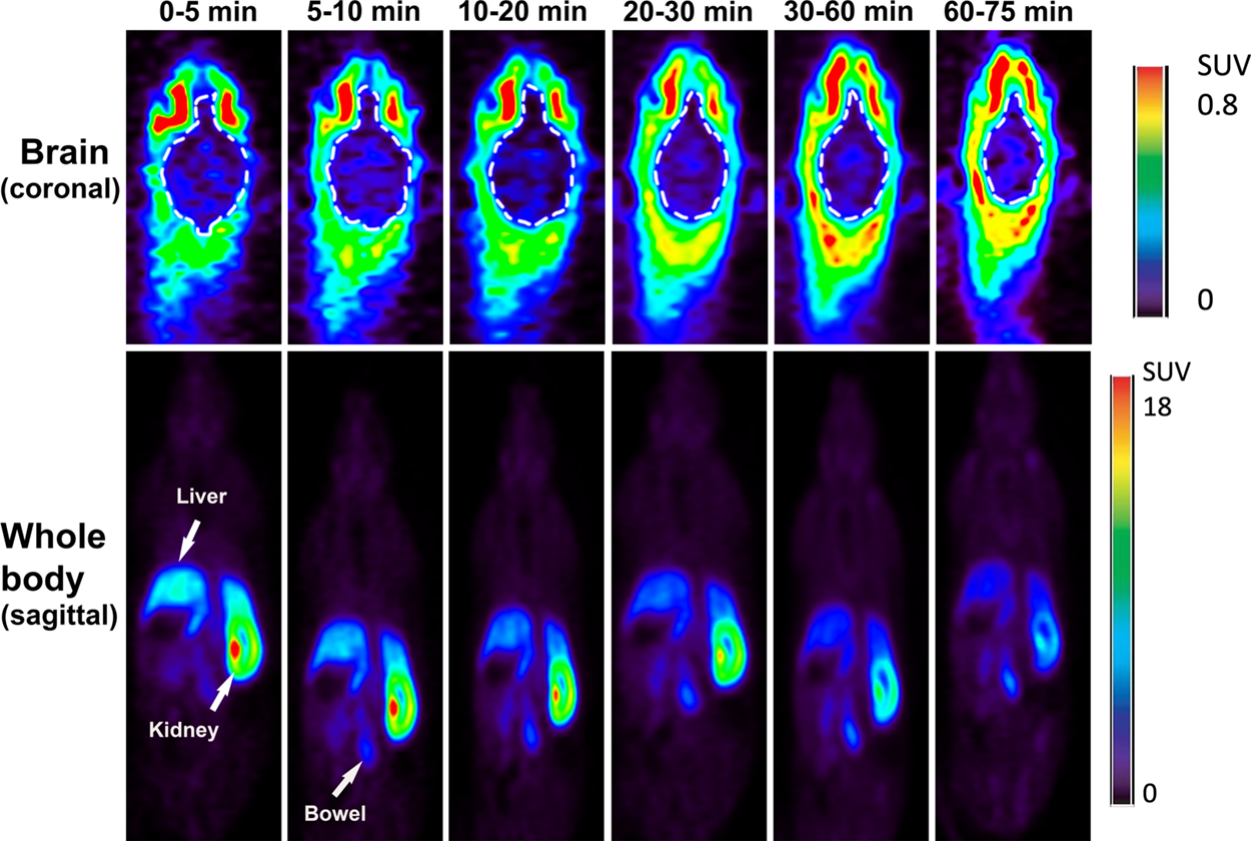
Dynamic PET imaging. Representative PET imaging data of 5XFAD mice (*n* = 2) describe time-course uptake in the brain (upper panel) and whole body (lower panel) immediately after intravenous injection of the [^18^F]PB0822 PET radioligand.

To test the time-dependent distribution to the brain, dynamic PET scans were obtained immediately after intravenous injection of 5XFAD mice (*n* = 2) with the [^18^F]PB0822 radioligand (400 μCi/0.1–0.2 mL). The data showed that the probe was distributed to the brain 5 min postintravenous injection (Figure 7). This early accumulation and retention of the probe is modest yet abundant enough for detection. The time-activity curve (TAC) data showed that the uptake in the cortex and cerebellum is higher than that in other regions, suggesting that QC activity might be more prominent in these brain subregions (Figure S19, SI). The whole-body PET imaging data indicated that imaging QC with this probe is unique because there was no indication of an overwhelming background signal from peripheral tissues and organs (Figure 7, lower panel). Aside from remarkable signals in the brain, there was also early detection of a strong signal in the kidneys, 5–10 min post injection, suggesting some QC activity in the kidneys (Figure 7, lower panel).

#### Copious Presence of Abeta-pE3 Found in the Brains of 5XFAD Mice

**Figure 8:**
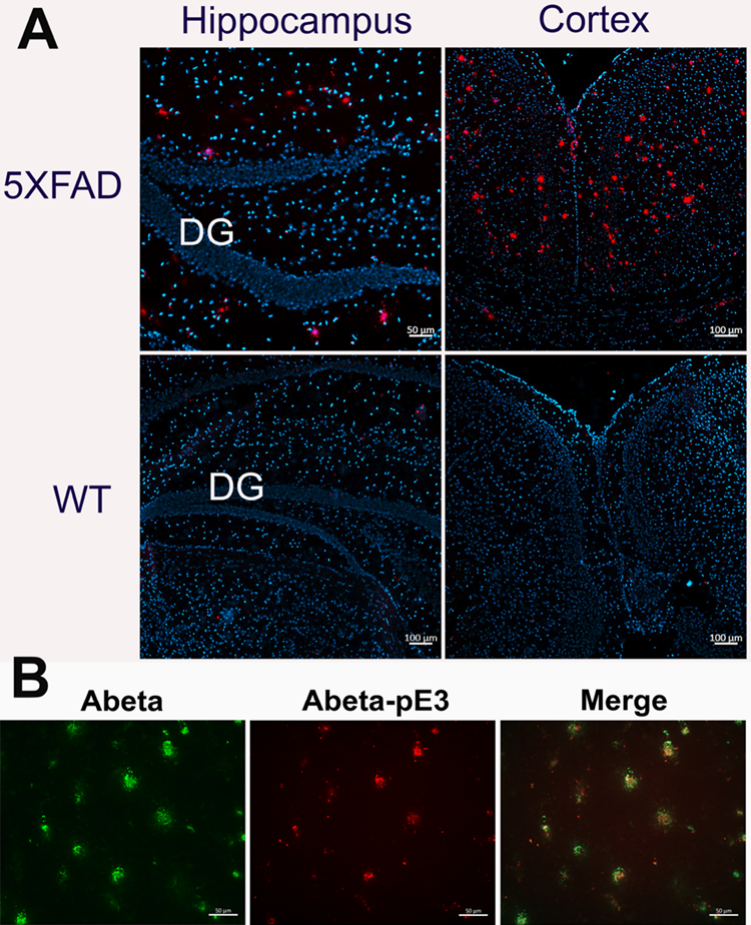
Immunohistochemistry to assess Abeta-pE3 levels in the brains. (A) Abeta-pE3 immunohistochemistry (red, 668 nm channel) of representative staining of Abeta-pE3 on DAPI (blue)-stained coronal brain sections (8–10 μm) using anti- Abeta-pE3 antibodies on WT vs 5XFAD mice (*n* = 3, each; four slides were observed per mouse). Quantitative pixel counts obtained for WT and 5XFAD are compared; (B) representative data of double immunostaining of Abeta and Abeta-pE3 on 5XFAD coronal brain slides (8–10 μm) and merged data. Quantitative pixel counts obtained for WT and 5XFAD are compared with significance, *p* < 0.05.

Coronal brain sections of approximately 8–10 μm thickness of WT (*n* = 3) and 5XFAD (*n* = 3) mice were stained with anti-Abeta-pE3 primary antibodies and visualized with a dye-labeled secondary antibody using a fluorescent microscope. The data indicated that there is no Abeta-pE3 in WT mouse brains (Figure 8). In contrast, 5XFAD brains harbored significant levels of Abeta-pE3 in the brain. More Abeta-pE3 was detected in the cortex compared with that in the hippocampus. This regional distribution of Abeta-pE3 is the product of QC activity, which was observed in the in vivo PET imaging data using [^18^F]PB0822.

## Conclusions

Inhibition of QC activity is an ideal target for treating AD. A recent study showed that treating mouse models of AD with oral doses of a QC inhibitor resulted in reduced pyroglutamate Abeta burden, diminished plaque formation, and improved cognition.^18^ Other work has shown that treating mice with anti-Abeta-pE3 monoclonal antibodies resulted in the attenuation of behavioral deficits and clearance of Abeta in preclinical mouse models.^38^ Taking all of these promising data into account, there is merit in developing imaging technology to help to assess these observations noninvasively. Furthermore, this probe will help to speed up the screening of QC inhibitors and evaluate the efficacy of these drugs in clinical trials. Since QC is involved in the early onset of AD, this probe can potentially help to stratify AD patients admitted to clinical trials; thus, it could hold considerable benefits for future AD diagnosis, prognosis, management, and treatment.

In summary, we report the development of [^18^F]PB0822 for imaging the QC activity in AD. This preclinical study suggests that this probe can be translated to humans. Aside from specificity for QC, the probe has an acceptable solubility profile, enabling the formulation for in vivo applications. Furthermore, [^18^F]PB0822 can cross the blood–brain barrier and pinpoint QC in the brains of 5XFAD mice. We did not observe adverse effects in animals during this pilot imaging work. Further safety analysis is underway, including cell-based toxicity and whole-body toxicology.

## Supporting Information Available

Detailed and complete chemical synthesis and characterization, including ^1^H NMR and ^13^C NMR for all intermediates and the standard compound of [^19^F]PB0822 and the tosylate precursor, HPLC chiral separation data for compound 4, HPLC data and conditions to confirm the identity of the QC PET [^18^F]PB0822 radioligand versus the standard compound, and TAC data to describe the regional specific uptake of [^18^F]PB0822 in the brain of a 5XFAD mouse (PDF)

## Abbreviations

PET: positron emission tomography
AD: Alzheimer’s disease
QC: glutaminyl cyclase
Abeta: amyloid-β
WT: wild-type
TMSCN: trimethylsilyl cyanide
Pd: palladium
CDI: 1,1′-carbonyldiimidazole
TBAF: tetra-*n*-butylammonium fluoride
K_2.2.2_: kryptofix 222
HPLC: high-performance liquid chromatography
EOS: end of synthesis
TAC: time–activity curve
NMR: nuclear magnetic resonance
IHC: immunohistochemistry
