# Supplementary material for: Synthesis and Evaluation of a Novel PET Radioligand for Imaging Glutaminyl Cyclase Activity as a Biomarker for Detecting Alzheimer’s Disease
Source: ACS Sens. 2024 May 8;9(5):2605–13. doi: 10.1021/acssensors.4c00313 (PMC11129349; doi:10.1021/acssensors.4c00313)
Supplement: Supplementary file 1 — se4c00313_si_001.pdf [file se4c00313_si_001.pdf]

Synthesis and evaluation of a novel PET radioligand for imaging glutaminyl cyclase activity as a biomarker for detecting Alzheimer's disease

William J. Behof [1,2], Justin R. Haynes [1,2], Clayton A. Whitmore [1,2], Yiu-Yin Cheung [1,2], Mohammed N. Tantawy [1,2], Todd E. Peterson [1,2,7], Printha Wijesinghe [3], Joanne A. Matsubara [3] and Wellington Pham [1,2,4,5,6,7,8,9*]

[1] Vanderbilt University Institute of Imaging Science, Vanderbilt University Medical Center, Nashville, TN 37232, USA
[2] Department of Radiology and Radiological Sciences, Vanderbilt University Medical Center, Nashville, TN 37232, USA
[3] Department of Ophthalmology and Visual Sciences, University of British Columbia, Vancouver, BC V5Z3N9, Canada; printha.wijesinghe@ubc.ca (P.W.); joanne.matsubara@ubc.ca (J.A.M.)
[4] Vanderbilt Brain Institute, Vanderbilt University, Nashville, TN 37232, USA
[5] Vanderbilt Memory and Alzheimer's Center, Vanderbilt University Medical Center, Nashville, TN 37212, USA
[6] Department of Biomedical Engineering, Vanderbilt University, Nashville, TN 37235, USA
[7] Vanderbilt Ingram Cancer Center, Nashville, TN 37232, USA
[8] Vanderbilt Institute of Chemical Biology, Vanderbilt University, Nashville, TN 37232, USA
[9] Vanderbilt Institute of Nanoscale Science and Engineering, Vanderbilt University, Nashville, TN 37235, USA

* Correspondence: wellington.pham@vumc.org

Table of contents

1. General synthesis information 2
2. Synthesis of compound 1 2
3. Synthesis of compound 2 4
4. Synthesis of compound 3 6
5. Synthesis of compound 4 8
6. Chiral separation data for tosylate compound 4 11
7. Synthesis of the standard [^{19}F]PB0822 (compound 8) starting from tosylate compound 12
8. Synthesis of compound 5 12
9. Synthesis of compound 6 14
10. Synthesis of compound 7 16
11. Synthesis of compound 8 18
12. Characterization of the [^{18}F]PB0822 radioligand 21
13. Time-activity curve (TAC) data of the dynamic uptake of [^{18}F]PB0822 23

General Synthesis

All commercially available reagents and solvents were used as received without further purification. All of the reaction progress were monitored by an Agilent LC/MS 1260 Infinity II. Products were purified using a Telodyne Combiflash Rf automated purification instrument using normal phase or reverse phase unless otherwise specified. ^{1}H- and ^{13}C-NMR spectra were recorded on a 600 MHz Bruker spectrometer equipped with a cryogenic radio frequency probe. ^{1}H-NMR chemical shifts were referenced to the residual solvent signal; 13C NMR chemical shifts were referenced to the deuterated solvent signal. Data are presented as follows: chemical shift d (ppm), multiplicity (s = singlet, d = doublet, dd = doublet of doublet, t = triplet, m = multiplet, dt = doublet of triplet).

Synthesis of 3-(4-formylphenoxy)propyl 4-methylbenzenesulfonate: Compound 1. To a stirring solution of 4-hydroxy benzaldehyde (1.0 g, 8.2 mmol), and 1,3 propane di-tosylate (6.3 g, 16.4 mmol), in acetonitrile (60 mL) was added K_2CO_3 (2.3 g, 16.4 mmol) and heated to 60°C overnight. This solution was cooled and diluted with H_2O and EtOAc. The product was extracted 3x with EtOAc. The organic layers were combined, washed with brine, dried over Na_2SO_4, filtered, and concentrated under reduced pressure. The product was purified by flash chromatography (0-100% CH_2Cl_2/(20%MeOH/CH_2Cl_2)) over 18 minutes to provide the final product as a white solid (2.5 g, 91% yield). ^{1}H $CDCl_3$ (400.13 mHz): 9.90 (s, 1H); 7.82 (d, J=8.8 Hz, 2H); 7.76 (d, 8.3 Hz, 1H); 7.25 (d, 8.2 Hz, 2H); 6.88 (d, 8.8 Hz, 2H); 4.26 (t, J=5.9 Hz, 2H); 4.05 (t, J=5.9 Hz, 2H); 2.38 (s, 3H); 2.16 (m, 2H). ^{13}C $CDCl_3$ (100.6 mHz): 190.7, 163.4, 144.9, 132.7, 131.9, 130.1, 129.8, 127.8, 114.6, 66.6, 63.4, 28.7, 21.6.
HRMS (ESI/Q-TOF) m/z: [M + H]+ Calcd for C17H18O5S 335.0948; Found 335.0948.

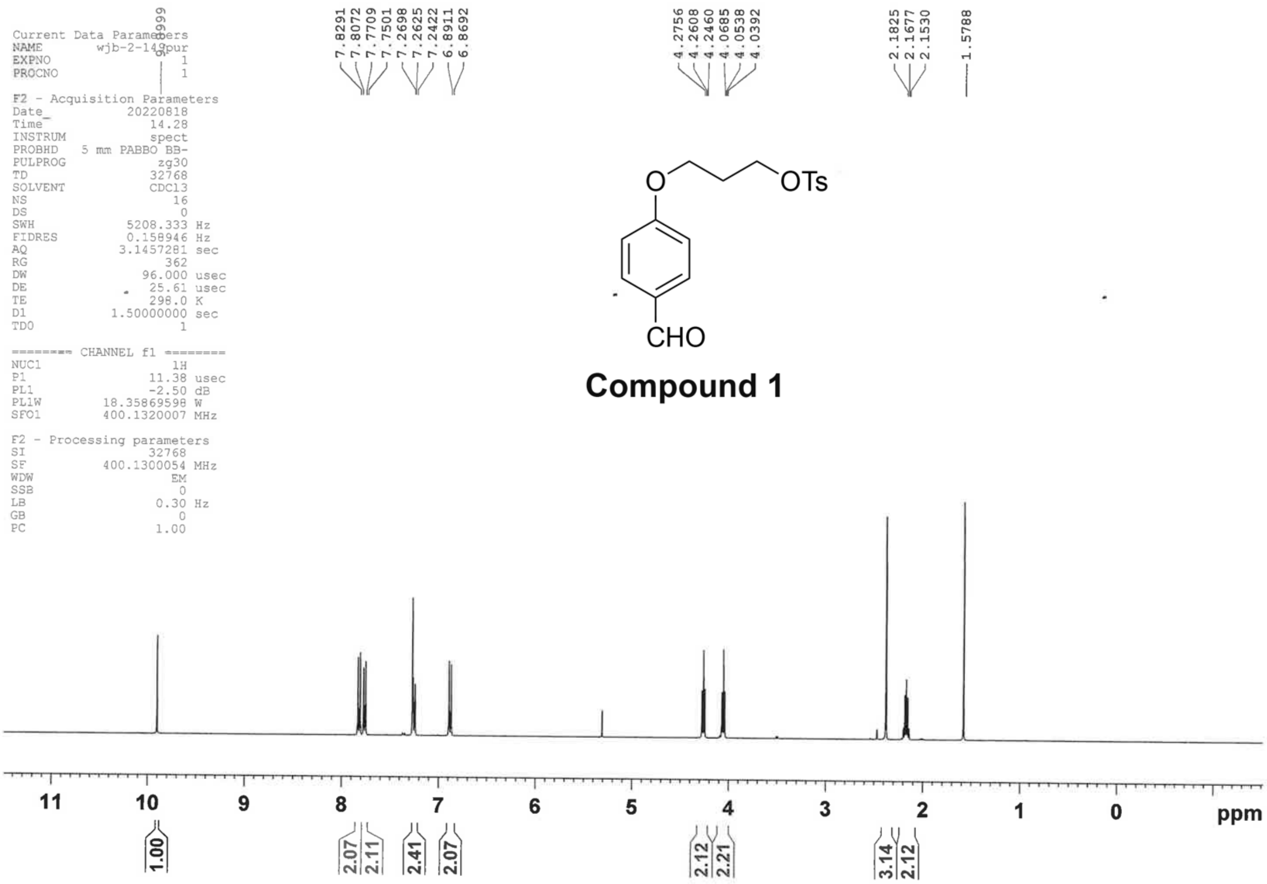

Figure S1. ^{1}H-NMR of compound 1 in chloroform-*d*

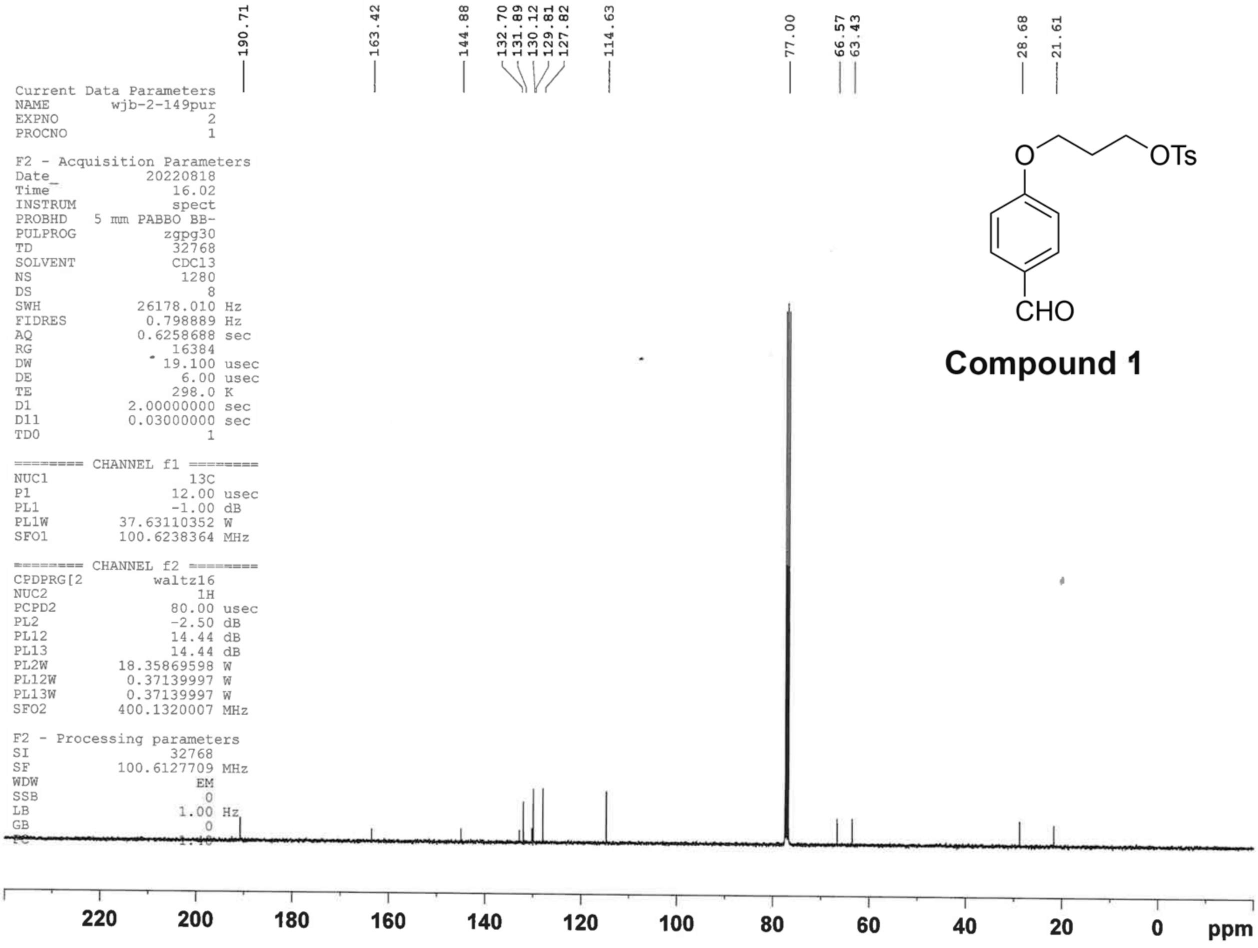

Figure S2. ^{13}C-NMR of compound 1 in chloroform-*d*

Synthesis of 3-(4-(((1*H*-benzo[*d*]imidazol-5-yl)amino)(cyano)methyl)phenoxy)propyl 4-methylbenzenesulfonate: Compound 2. To a stirring solution of **1** (1.0 g, 2.9 mmol) and 2-amino benzimidazole (464 mg, 3.5 mmol) in CH_2Cl_2 (3 mL) and MeOH (3 mL) was added trimethylsilyl cyanide (575 mg, 5.8 mmol). This solution was stirred overnight at room temperature. The reaction was concentrated under reduced pressure. The residue was diluted with H_2O and EtOAc. The product was extracted 3x with EtOAc. The organic layers were combined, washed with brine, dried over Na_2SO_4, filtered, and concentrated under reduced pressure. The product was purified by flash chromatography (0-100% CH_2Cl_2/(20%MeOH/CH_2Cl_2)) over 18 minutes to afford a final product as dark brown solid, (900 mg, 65% yield). Note: to obtain a pure NMR, the sample was purified by flash chromatography. However, the material decomposes while sitting in MeOH, so for higher yields in subsequent steps the

reaction was used as is. ^{1}H MeOD (600.13 mHz): 8.01 (s, 1H); 7.71 (d, J=8.3 Hz, 2H); 7.51 (d, J=8.6 Hz, 2H); 7.45 (d, J=8.7 Hz, 1H); 7.27 (d, J=8.0 Hz, 2H); 7.00 (d, J=2.0 Hz, 1H); 6.87 (dd, J_1=8.7 Hz, J_2=2.2 Hz); 6.84 (d, J=8.7 Hz, 2H); 5.67 (s, 1H), 4.22 (t, J=6.1 Hz, 2H); 3.93 (t, J=5.8 Hz, 2H); 2.32 (s, 3H); 2.08 (m, 2H). ^{13}C MeOD (150.9 mHz): 160.5, 146.5, 144.0, 141.4, 134.0, 131.7, 131.1, 129.7, 129.1, 128.9, 128.5, 120.5, 115.9, 115.8, 115.7, 114.4, 68.4, 64.3, 51.0, 29.8, 21.6.
HRMS (ESI/Q-TOF) m/z: [M + H]+ Calcd for C25H24N4O4S 477.1591; Found 477.1588.

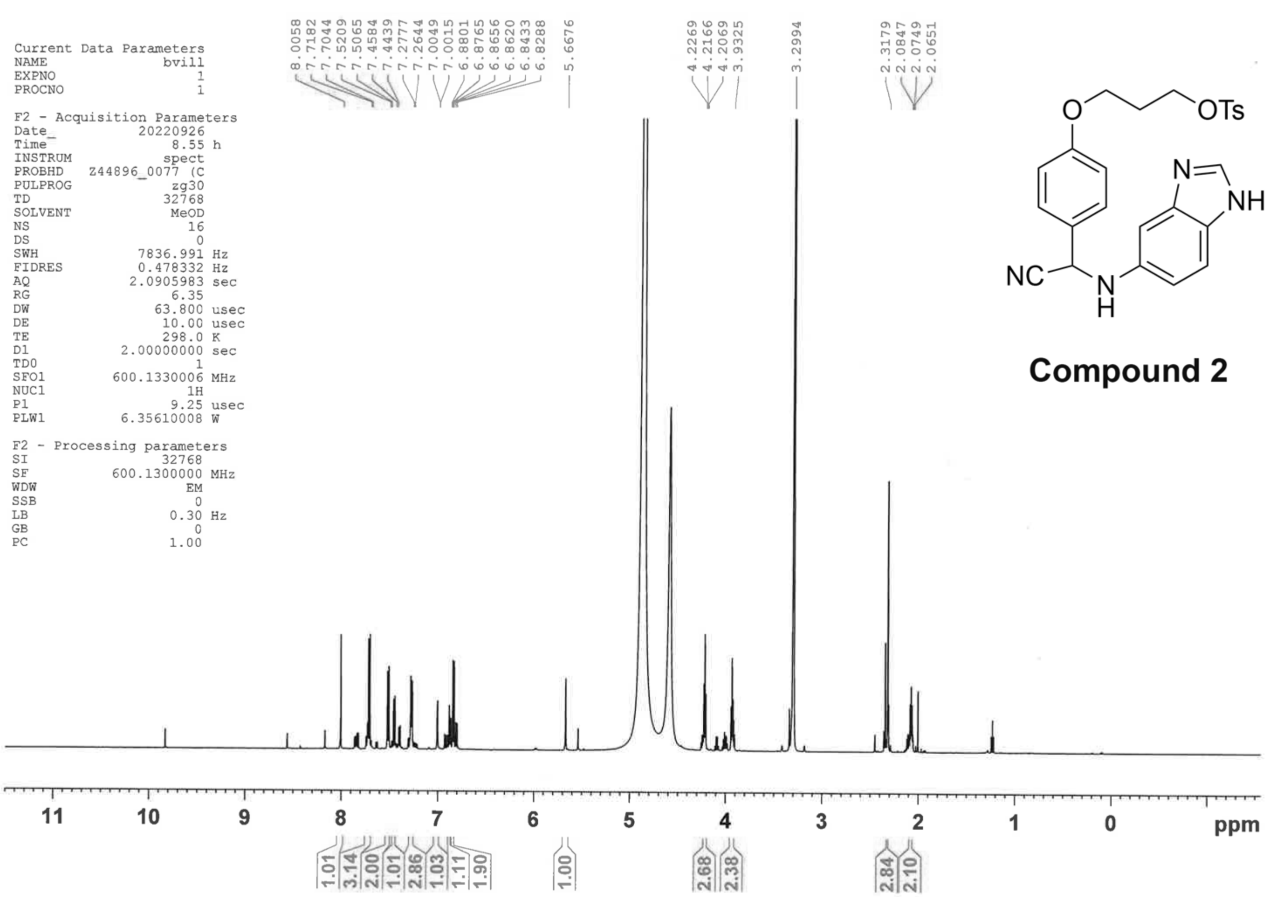

Figure S3. ^{1}H-NMR of compound 2 in methanol-*d*

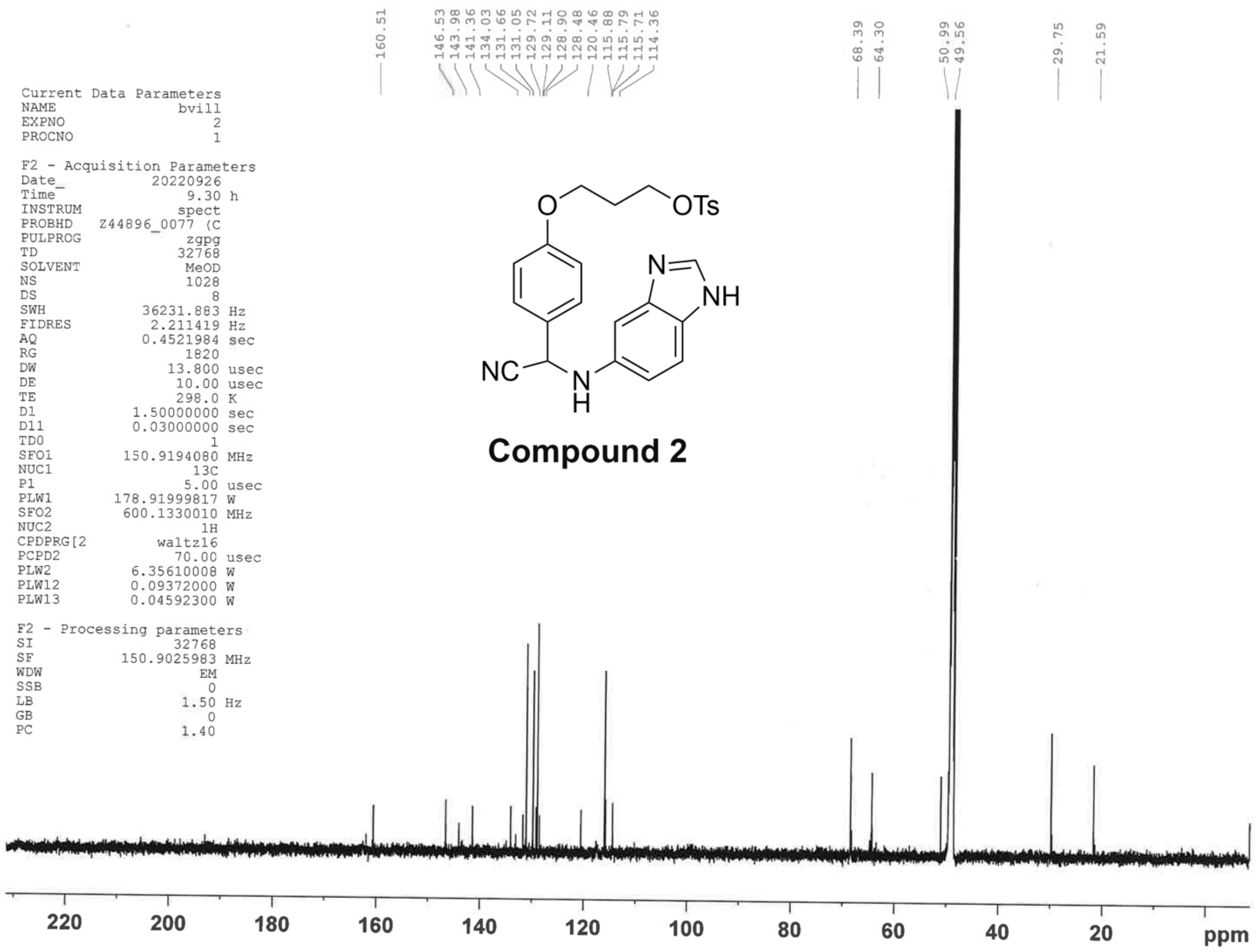

Figure S4. ^{13}C-NMR of compound 2 in methanol-*d*

Synthesis of 3-(4-(1-((1*H*-benzo[*d*]imidazol-5-yl)amino)-2-aminoethyl)phenoxy)propyl 4-methylbenzenesulfonate: Compound 3. To a stirring solution of **2** (900 mg, 1.9 mmol) in acetic acid (15 mL) in a Parr hydrogen bomb was added 10% wet Pd/C (200 mg, 1.9 mmol). The vessel was purged of air in the following way: placed under vacuum and refilled with 130 psi of hydrogen three times. The solution was stirred overnight. Reaction monitoring indicated it was 50% complete, so an addition of equivalent of 10% wet Pd/C (200 mg, 1.9 mmol) was added. The vessel was recharged and purged with 130 psi of hydrogen three times and let stir overnight. Upon completion, the reaction was filtered over celite, washed with MeOH, and concentrated under reduced pressure; acetonitrile was added to help remove the remaining acetic acid. The product was purified by flash chromatography (0-100% CH_2Cl_2/(20%MeOH/CH_2Cl_2)) over 18 minutes to afford a final product as a dark brown solid (720

mg, 79% yield). ^{1}H DMSO (600.13 mHz): 9.21 (s, 1H); 8.08 (br.s, 3H); 7.72 (d, J=8.3 Hz, 2H); 7.55 (d, J=8.9 Hz, 2H); 7.33 (m, 4H); 6.95 (dd, J1=8.9 Hz, J2=2.0 Hz, 1H); 6.80 (d, J=8.8 Hz, 1H); 6.65 (d, J=1.9 Hz, 1H); 4.70 (m, 1H); 4.13 (m, 2H); 3.86 (m, 2H); 3.11 (m, 2H); 2.28 (s, 3H); 1.97 (m, 2H). ^{13}C DMSO (150.9 mHz): 158.7, 158.5, 158.3, 158.0, 157.8, 146.4, 144.9, 138.0, 132.1, 132.0, 131.5, 130.1, 128.1, 127.8, 127.5, 125.5, 122.5, 117.1, 115.7, 115.1, 94.2, 67.6, 63.1, 54.6, 44.4, 40.4, 28.1, 21.0. HRMS (ESI/Q-TOF) m/z: [M + H]+ Calcd for C25H28N4O4S 481.1904; Found 481.1929.

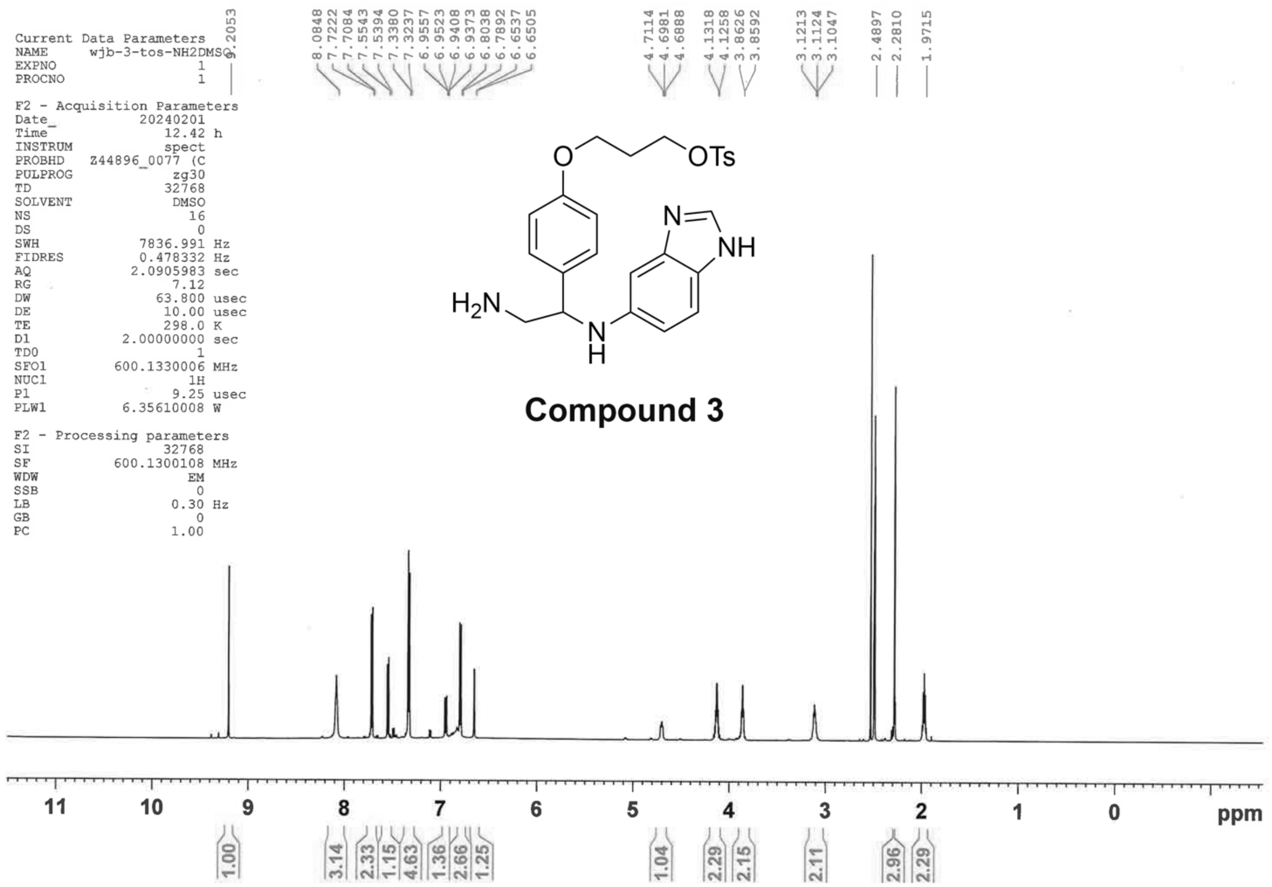

Figure S5. ^{1}H-NMR of compound 3 in DMSO-*d*

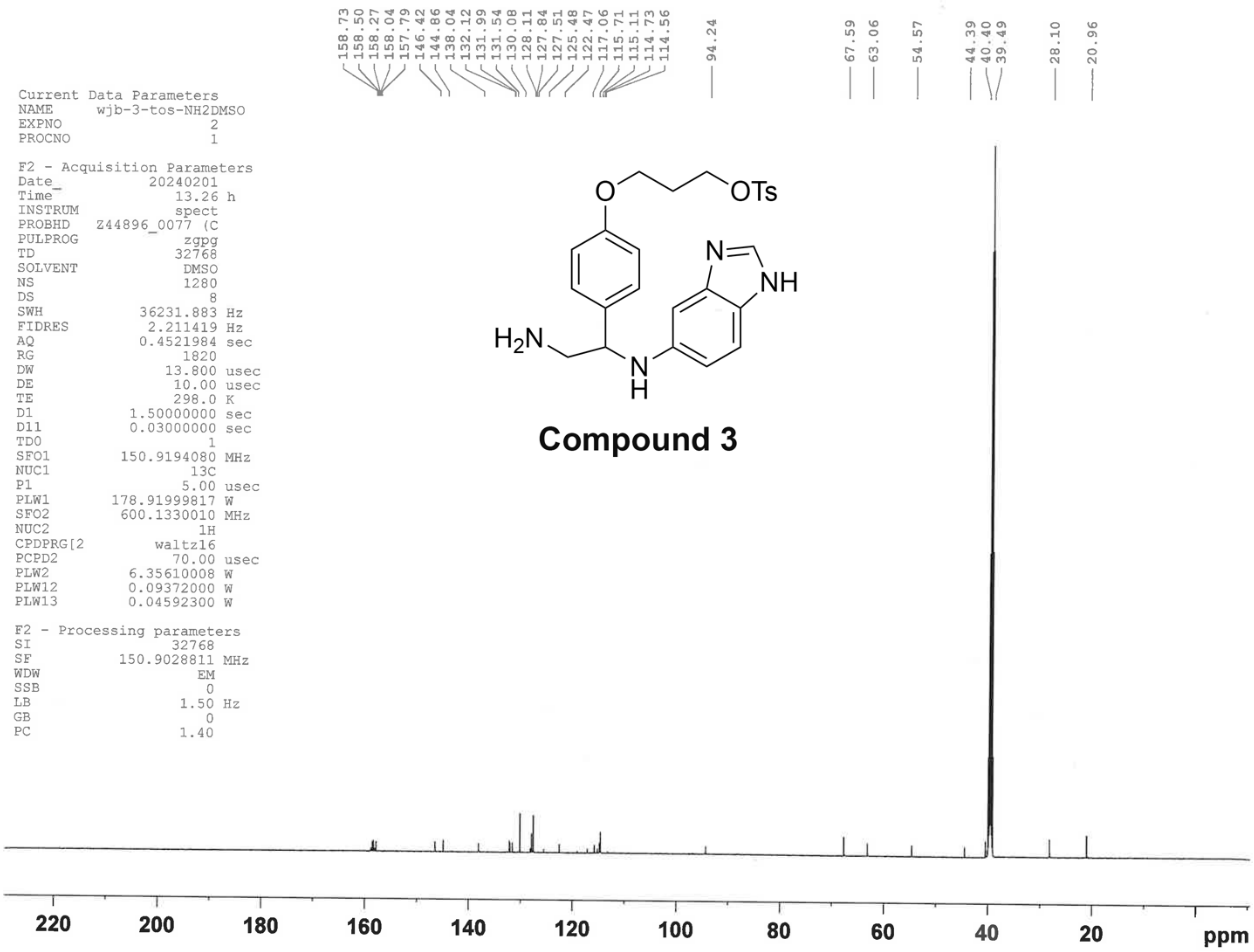

Figure S6. ^{13}C-NMR of compound 3 in DMSO-*d*

Synthesis of 3-(4-(3-(1*H*-benzo[*d*]imidazol-5-yl)-2-oxoimidazolidin-4-yl)phenoxy)propyl 4-methylbenzenesulfonate: Compound 4. To a stirring solution of **3** (720 mg, 1.5 mmol), triethylamine (1.5 g, 15 mmol) in THF (50 mL) was added 1,1'-Carbonyldiimidazole (243 mg, 1.5 mmol). The solution was stirred at room temperature overnight. This solution was diluted with H_2O and CH_2Cl_2. The product was extracted 3x with CH_2Cl_2. The organic layers were combined, washed with brine, dried over Na_2SO_4, filtered, and concentrated under reduced pressure. The temperature was kept under 30°C so as to prevent the substitution by imidazole. The product was purified by reverse phase chromatography (Gilson, Phenomenex C18 (00B-4454-DO-AX) Gemini-NX 5u, 110 A, 50 x 21.20mm column), 98% H_2O/2% ACN to 100% ACN over 8 minutes to afford the final product as a white solid (170 mg, 23%

yield). ^{1}H MeOD (600.13 mHz): 9.05 (s, 1H); 7.85 (s, 1H); 7.66 (d, J=8.28 Hz, 2H); 7.63 (s, 1H); 7.62 (d, J=1.74 Hz, 1H); 7.29 (dd, J_1=6.84 Hz, J_2=6.42 Hz, 2H); 7.20 (d, J=7.98 Hz, 2H); 6.73 (d, J=1.92 Hz, 2H); 5.51 (dd, J_1=9.12 Hz, J_2=6.42 Hz, 1H); 4.14 (m, 2H), 3.98 (t, J=9.12 Hz, 1H); 3.86 (m, 2H); 3.34 (dd, J_1=9.12 Hz, J_2=6.30 Hz, 2H); 2.01 (m, 2H). ^{13}C MeOD (150.9 mHz): 162.0, 160.0, 146.3, 141.0, 139.2, 133.4, 132.8, 131.0, 128.94, 128.91, 128.6, 122.0, 116.0, 115.2, 107.5, 68.4, 64.3, 61.8, 48.2, 29.8, 21.5.

HRMS (ESI/Q-TOF) m/z: [M + H]+ Calcd for C26H26N4O5S 507.1697; Found 507.1687.

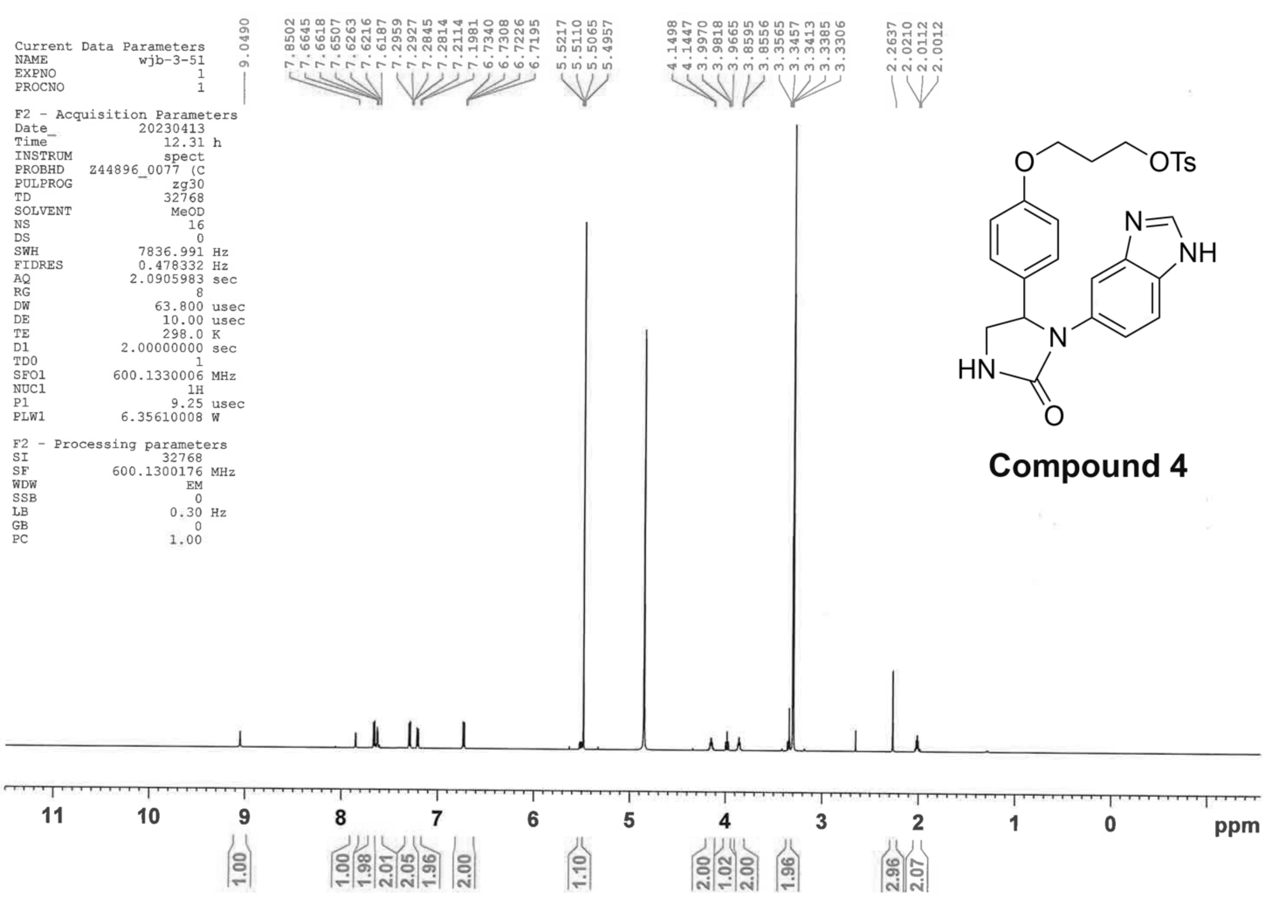

Figure S7. ^{1}H-NMR of compound 4 in methanol-*d*

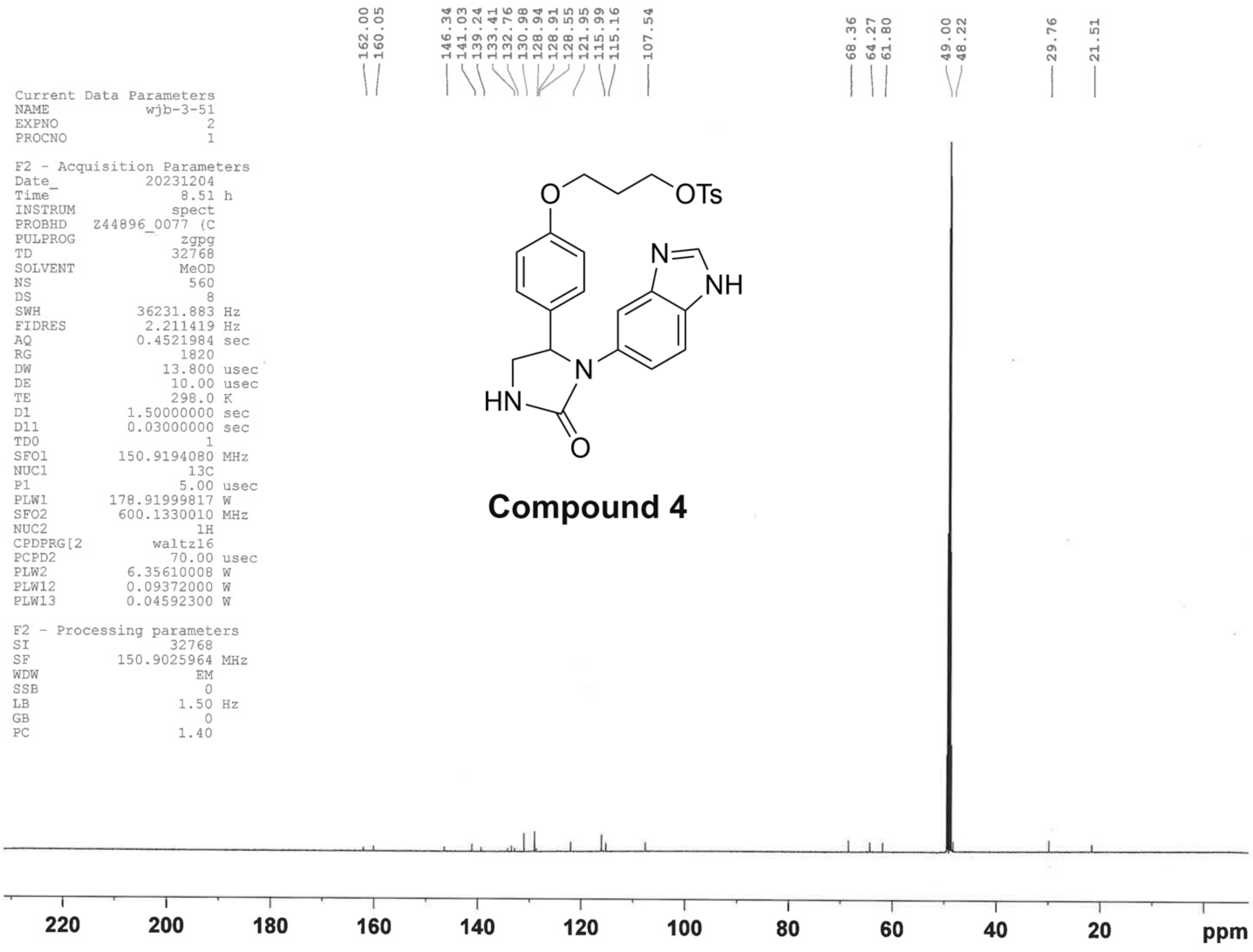

Figure S8. ^{13}C-NMR of compound 4 in methanol-*d*

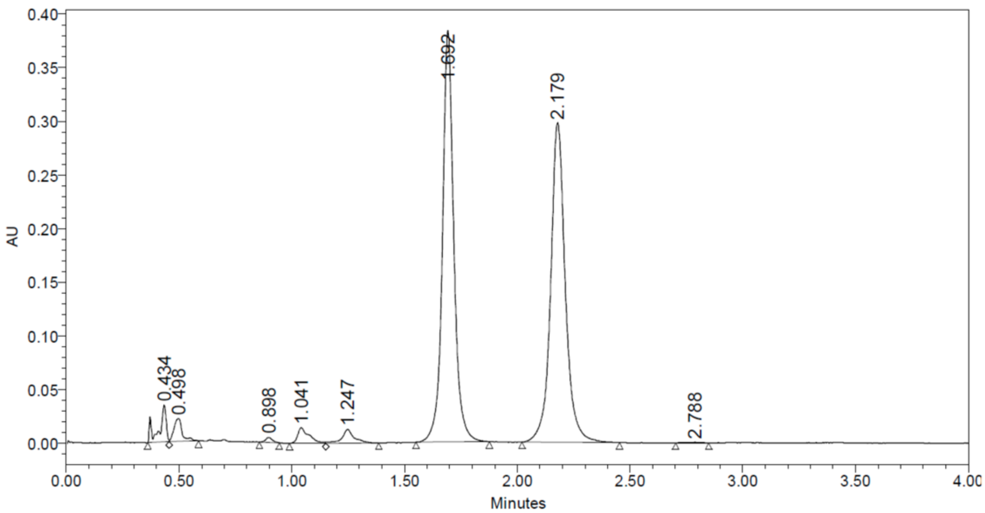

	RT	Area	% Area	Height
1	0.434	73991	2.53	34311
2	0.498	53979	1.84	21155
3	0.898	9012	0.31	4593
4	1.041	49898	1.70	14534
5	1.247	46653	1.59	12680
6	1.692	1345487	45.95	383373
7	2.179	1346304	45.98	297767
8	2.788	2855	0.10	629

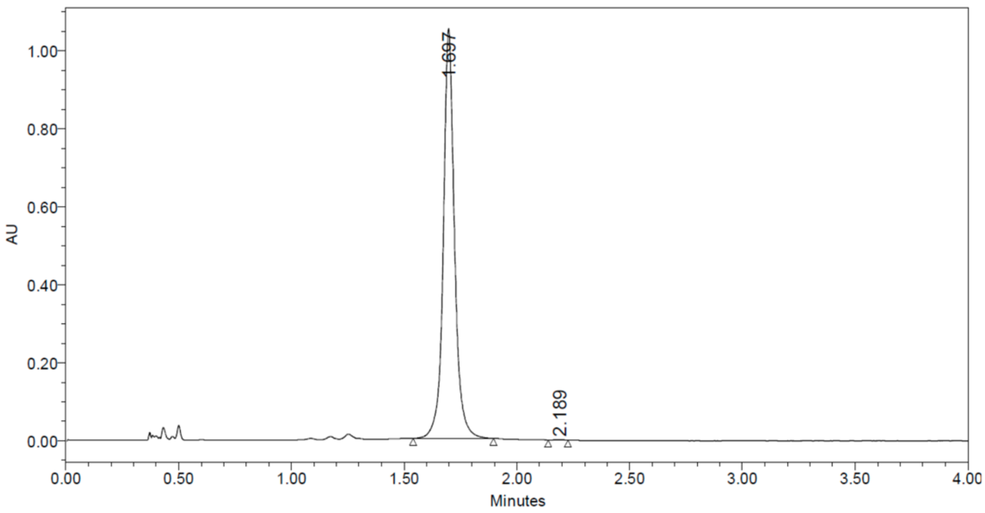

	RT	Area	% Area	Height
1	1.697	3621540	99.94	1049833
2	2.189	2058	0.06	894

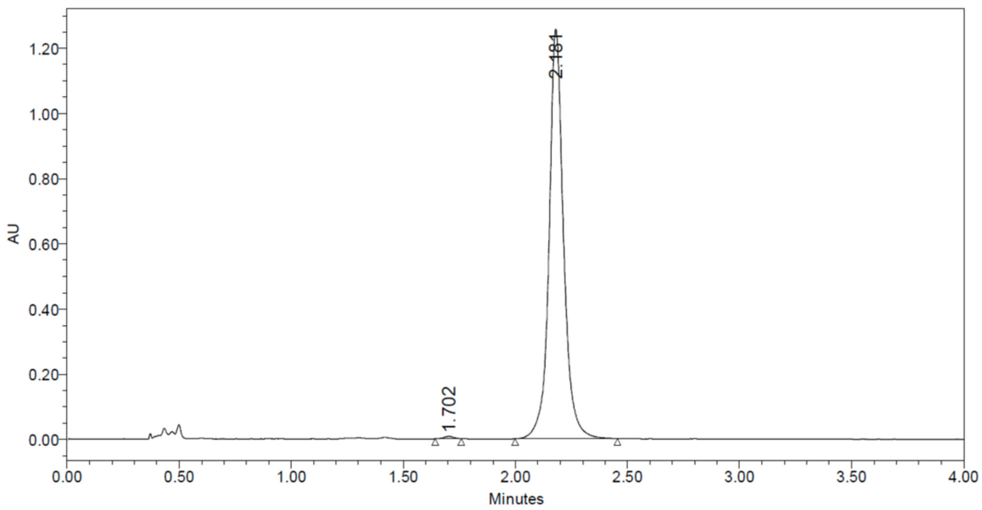

	RT	Area	% Area	Height
1	1.702	21313	0.38	7276
2	2.181	5618871	99.62	1255800

Figure S9. Chiral separation data for tosylate compound 4.

Synthesis of the standard [^{19}F]PB0822 (compound 8) starting from tosylate compound 4. To a stirring solution of 4 (15 mg, 0.03 mmol) in 1mL of THF was added TBAF (1.0 M in THF) (0.200 mL) and was stirred for 4 hours until completion. The resulting solution was concentrated under reduced pressure. The product was purified by reverse phase chromatography (Gilson, Phenomenex C18 (00B-4454-DO-AX) Gemini-NX 5u, 110 A, 50 x 21.20mm column), 98% H_2O/2% ACN to 60% ACN over 8 minutes to provide a final product with 45% yield.

Synthesis of 4-(3-fluoropropoxy)benzaldehyde: Compound 5. To a stirring solution of 4-hydroxy benzaldehyde (1.0 g, 8.1 mmol) and 1-iodo, 3-fluoro propane (1.54 g, 8.1 mmol) in acetonitrile (50 mL) was added K_2CO_3 (1.3 g, 9.7 mmol) and heated to 60°C overnight. The reaction was concentrated under reduced pressure. The residue was resuspended in CH_2Cl_2 and filtered. The supernatant was concentrated under reduced pressure and purified by flash chromatography. The product was purified by flash chromatography (0-100% CH_2Cl_2/(20%MeOH/CH_2Cl_2)) over 15 minutes to provide the final product as a clear oil (1.36 g, 92% yield). ^{1}H $CDCl_3$ (400.13 mHz): 9.87 (s, 1H); 7.82 (d, J=8.0 Hz, 2H); 6.99 (d, J=8.0 Hz, 2H); 4.64 (dt, J_1=48.0 Hz, J_2=5.7 Hz, 2H); 4.17 (t, J=6.1 Hz, 2H); 2.20 ((m, 2H). ^{13}C $CDCl_3$ (100.6 mHz): 190.7, 163.7, 131.9, 130.0, 114.7, 80.3 (d, J=165.0 Hz), 63.8 (d, J=5.0 Hz), 30.2 (d, J=20.1 Hz).
HRMS (ESI/Q-TOF) m/z: [M + H]+ Calcd for C10H11FO2 183.0816; Found 183.0795.

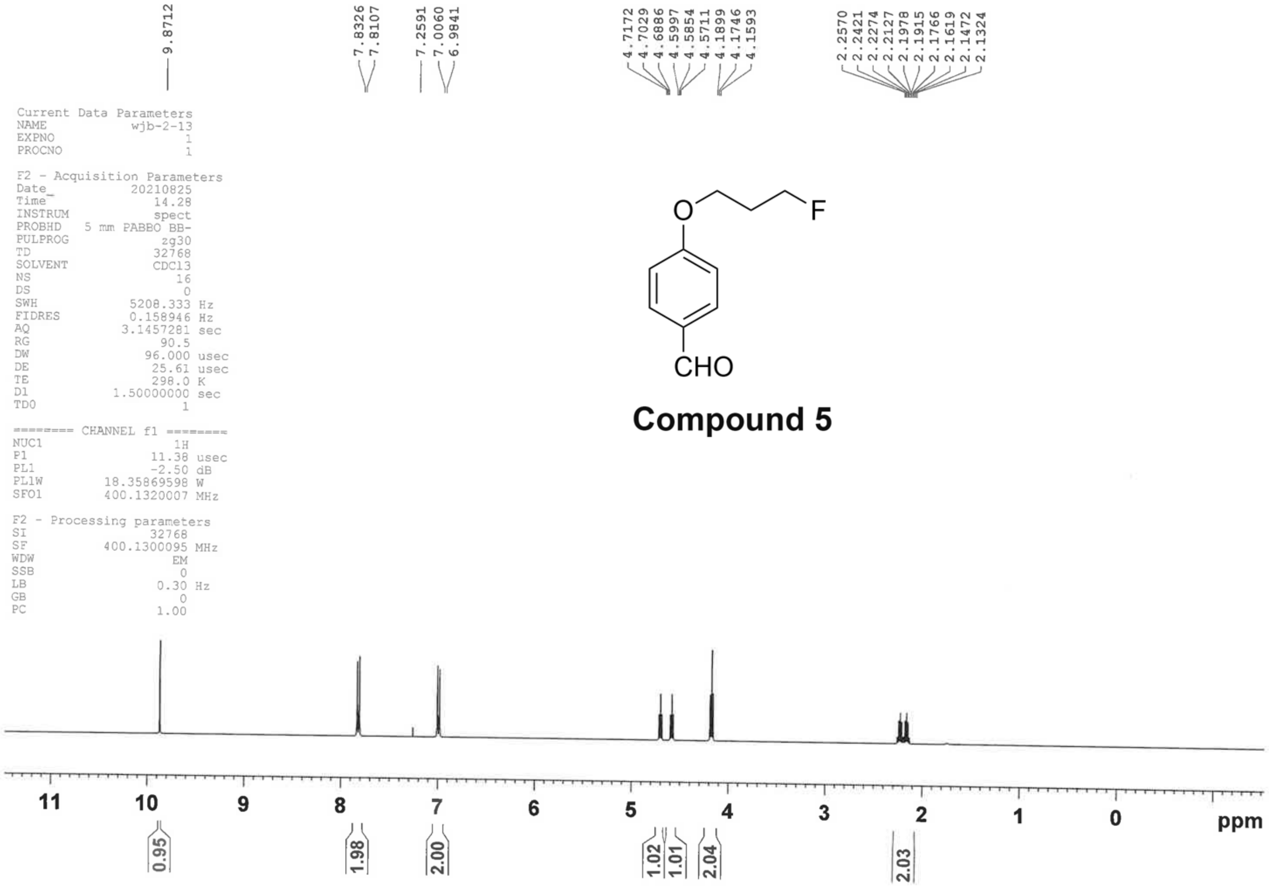

Figure S10. ^{1}H-NMR of compound 5 in chloroform-*d*

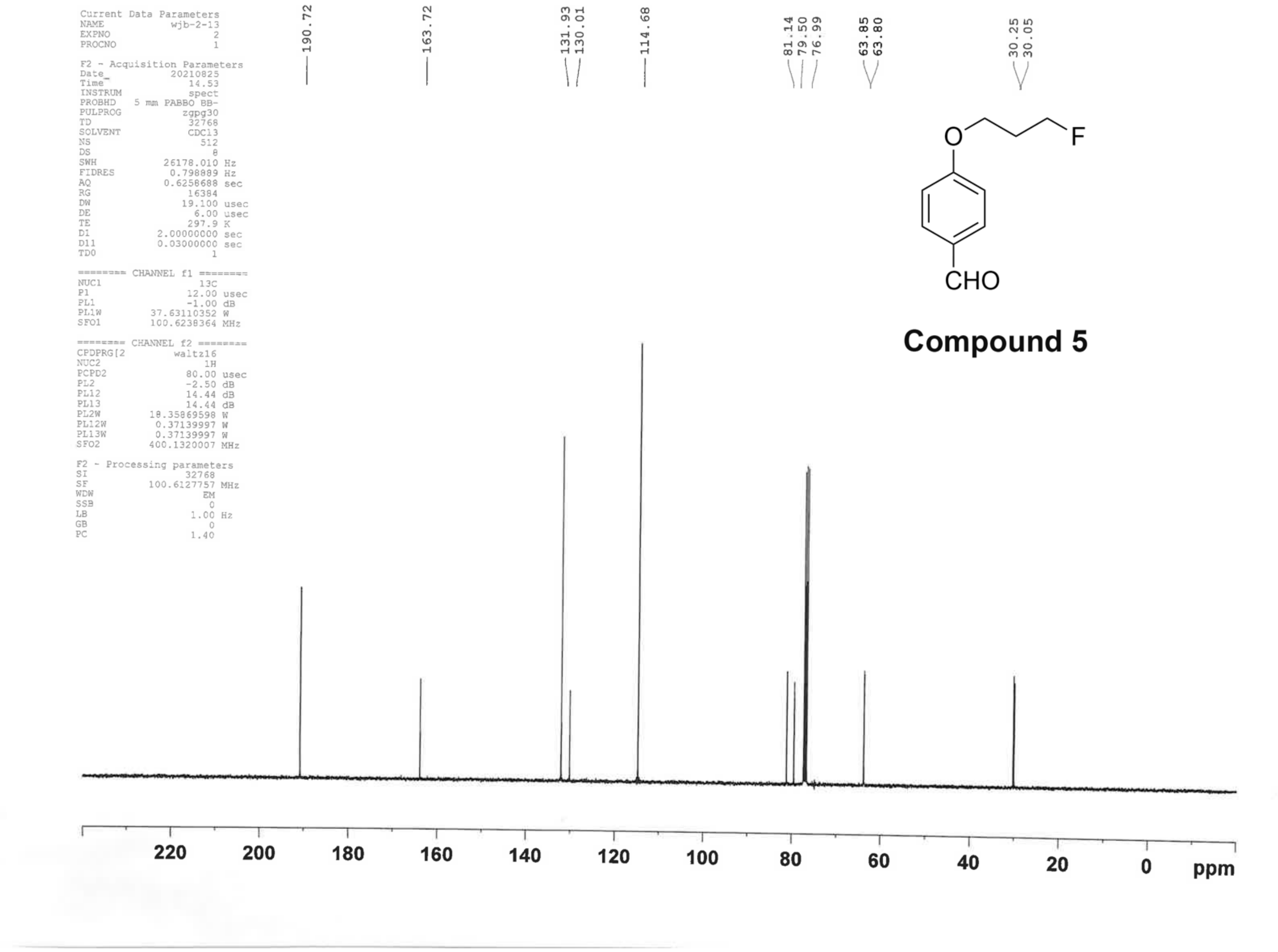

Figure S11. ^{13}C-NMR of compound 5 in chloroform-*d*

Synthesis of 2-((1*H*-benzo[*d*]imidazol-5-yl)amino)-2-(4-(3-fluoropropoxy)phenyl)acetonitrile: Compound 6. To a stirring solution of 5 (988 mg, 5.4 mmol) and 2-amino benzimidazole (722 mg, 5.4 mmol) in MeOH (3 mL) was added trimethylsilyl cyanide (642 mg, 6.5 mmol). This solution was stirred overnight at room temperature. The reaction was quenched with saturated bicarbonate and extracted 3x with CH_2Cl_2. The organic layers were combined, washed with brine, dried over Na_2SO_4, filtered, and concentrated under reduced pressure. The product was purified by flash chromatography (0-100% CH_2Cl_2/(20%MeOH/CH_2Cl_2)) over 30 minutes, (598 mg, 34% yield). Note: to obtain a pure NMR, the sample was purified by flash chromatography. However, the material decomposes while sitting in

MeOH, so for higher yields in subsequent steps, after reaction completion, it was concentrated and triturated with acetonitrile. The resulting slurry was filtered and used as a dark brown solid. ^1H $CDCl_3$ (600.13 mHz): 7.95 (s, 1H); 7.58 (br.s, 1H); 7.53 (d, J=5.8 Hz, 2H); 6.99 (m, 3H); 6.76 (dd, J_1=5.8 Hz, J_2=1.5 Hz, 1H); 5.34 (d, J=5.0 Hz, 1H); 4.66 (dd, J_1=31.4 Hz, J_2=3.8 Hz, 2H); 4.13 (t, J=4.0 Hz, 2H); 4.04 (d, J=5.3 Hz, 1H); 2.20 (m, 2H). ^{13}C $CDCl_3$ (150.9 mHz): 159.6, 141.5, 139.8, 130.5, 128.7, 126.1, 118.5, 115.2, 114.7, 112.9, 80.5 (d, J=164.5 Hz), 63.7 (d, J=4.5 Hz), 50.7, 30.3 (d, 19.6 Hz).
HRMS (ESI/Q-TOF) m/z: [M + H]+ Calcd for C18H17FN4O 325.1459; Found 325.1470.

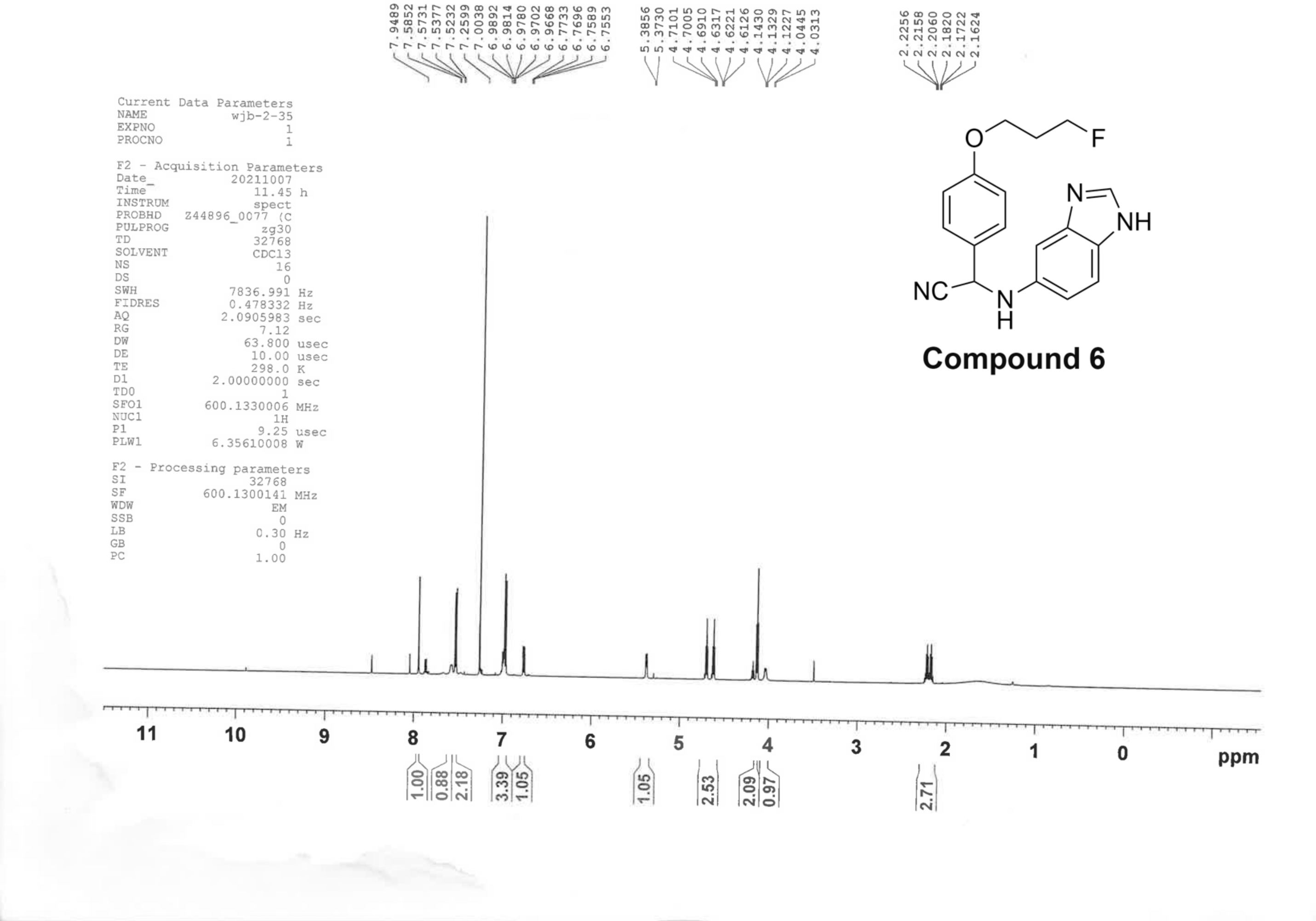

Figure S12. ^{1}H-NMR of compound 6 in chloroform-*d*

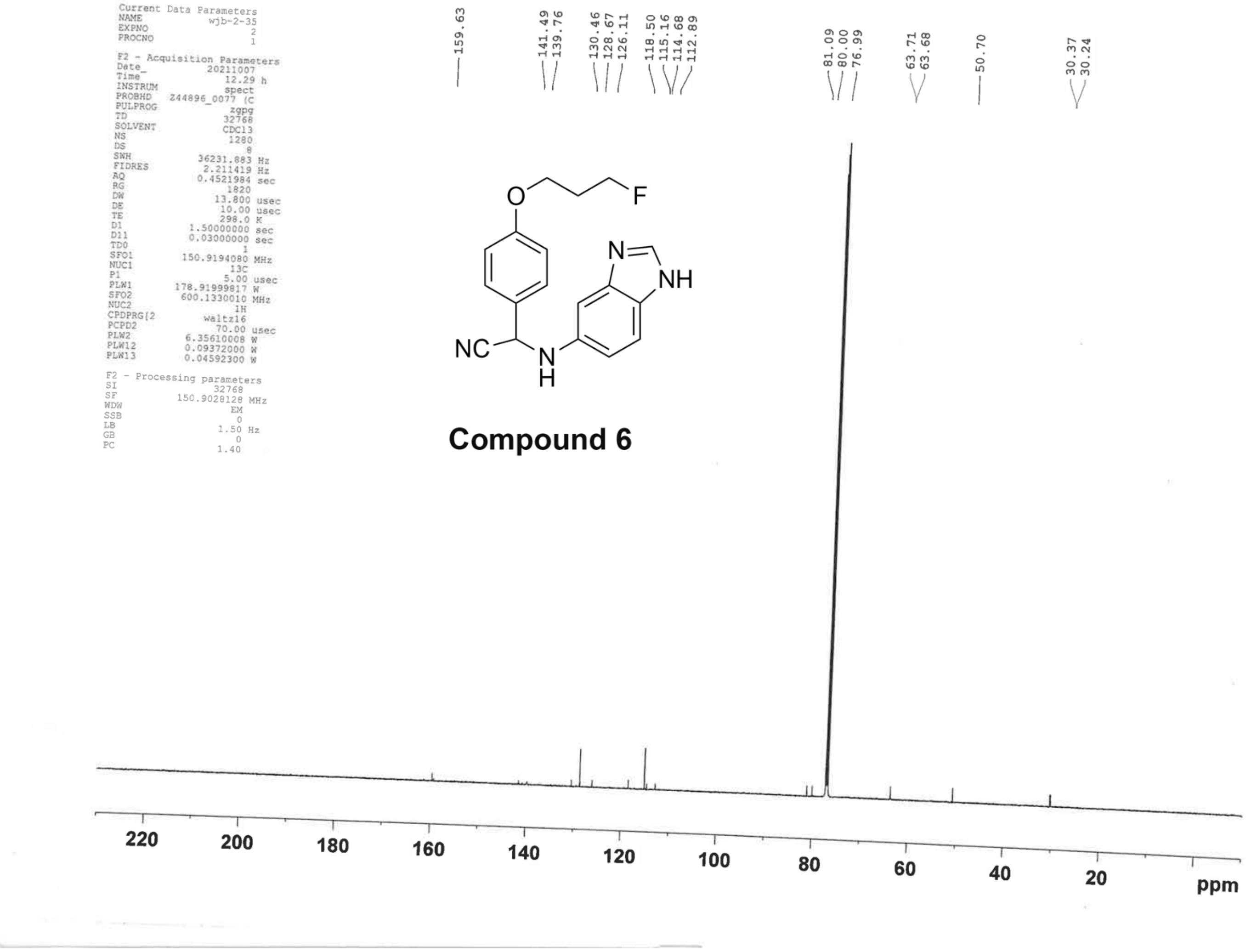

Figure S13. ^{13}C-NMR of compound 6 in chloroform-*d*

Synthesis of *N*[1]-(1*H*-benzo[*d*]imidazol-5-yl)-1-(4-(3-fluoropropoxy)phenyl)ethane-1,2-diamine: Compound 7. A stirring solution of **6** (252 mg, 0.78 mmol) in THF (10 mL) was cooled to -78°C. To this solution was added 1M DIBAL in THF dropwise (6.2 mL, 6.2 mmol). The reaction was allowed to warm to 0°C and stirred for 2 hrs. The reaction was quenched with 400uL of H_2O and 400uL of 15% NaOH and allowed to warm to room temperature. After 15 minutes, 1 mL of H_2O was added and was stirred for an additional 15 minutes. This slurry was then filtered over celite and washed with MeOH. The supernatant was concentrated under reduced pressure. The product was purified by flash chromatography (0-100% CH_2Cl_2/(30%MeOH/CH_2Cl_2)) over 18 minutes to afford a final product as a

dark brown solid (20 mg, 8% yield). ^{1}H MeOD (400.13 mHz): 7.82 (s, 1H), 7.31 (d, J=8.5 Hz, 2H); 6.86 (d, J=8.5 Hz, 3H); 6.86 (d, J=8.5 Hz, 2H); 6.74 (dd, J_1=8.7 Hz, J_2=1.9 Hz, 1H); 6.58 (s, 1H); 4.57 (dt, J_1=47.2, J_2=5.9 Hz, 2H); 4.36 (t, J=6.5 Hz, 1H);4.02 (t, J=6.2 Hz, 2H); 2.08 (m, 2H). ^{13}C MeOD (100.6 mHz): 159.5, 146.3, 140.3, 135.7, 128.9, 115.6, 113.9, 81.7 (d, J=163.0 Hz), 64.8 (d, 6.0 Hz), 61.7, 49.6, 31.5 (d, 20.1 Hz).
HRMS (ESI/Q-TOF) m/z: [M + H]+ Calcd for C18H21FN4O 329.1772; Found 329.1770.

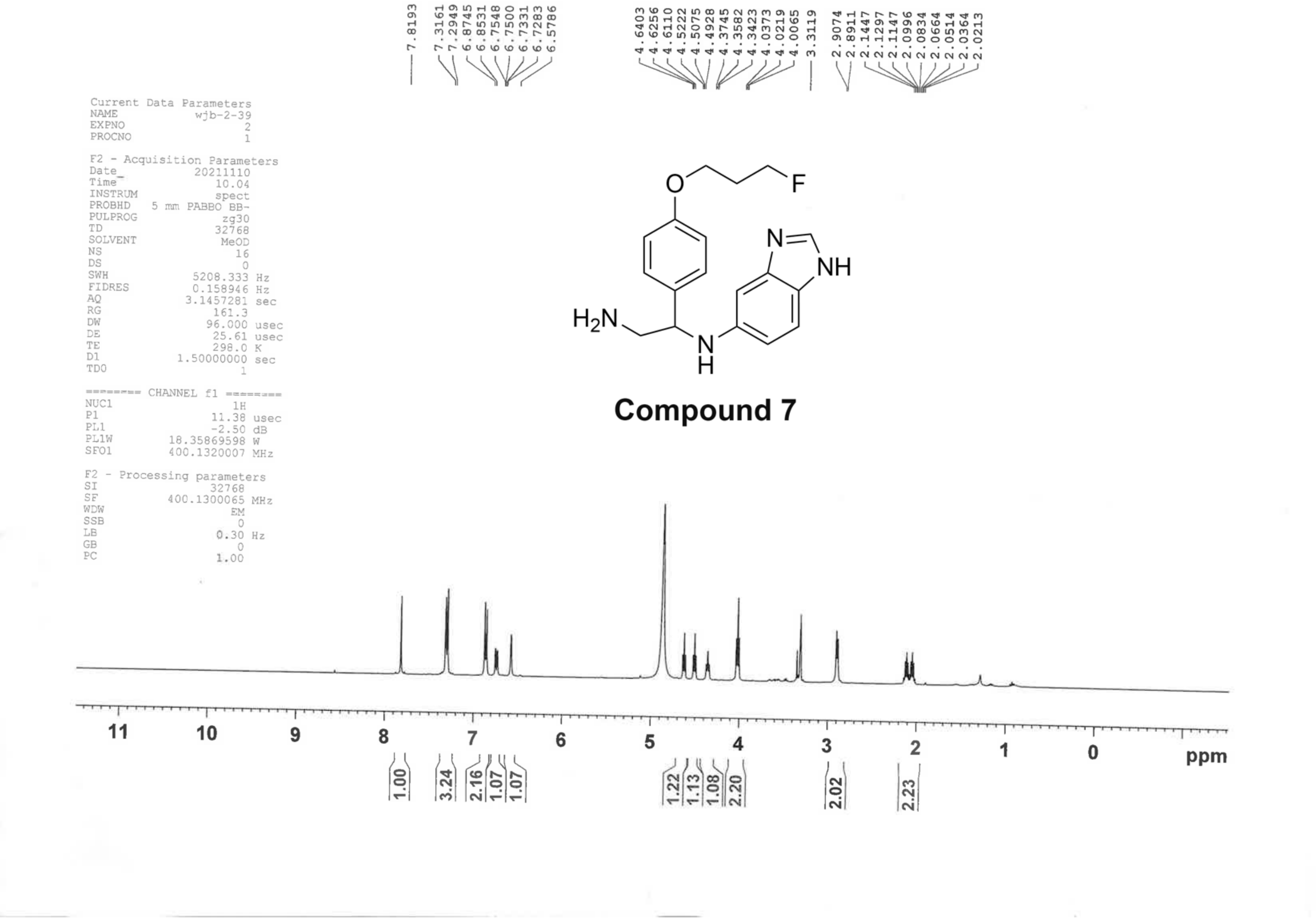

Figure S14. ^{1}H-NMR of compound 7 in methanol-*d*

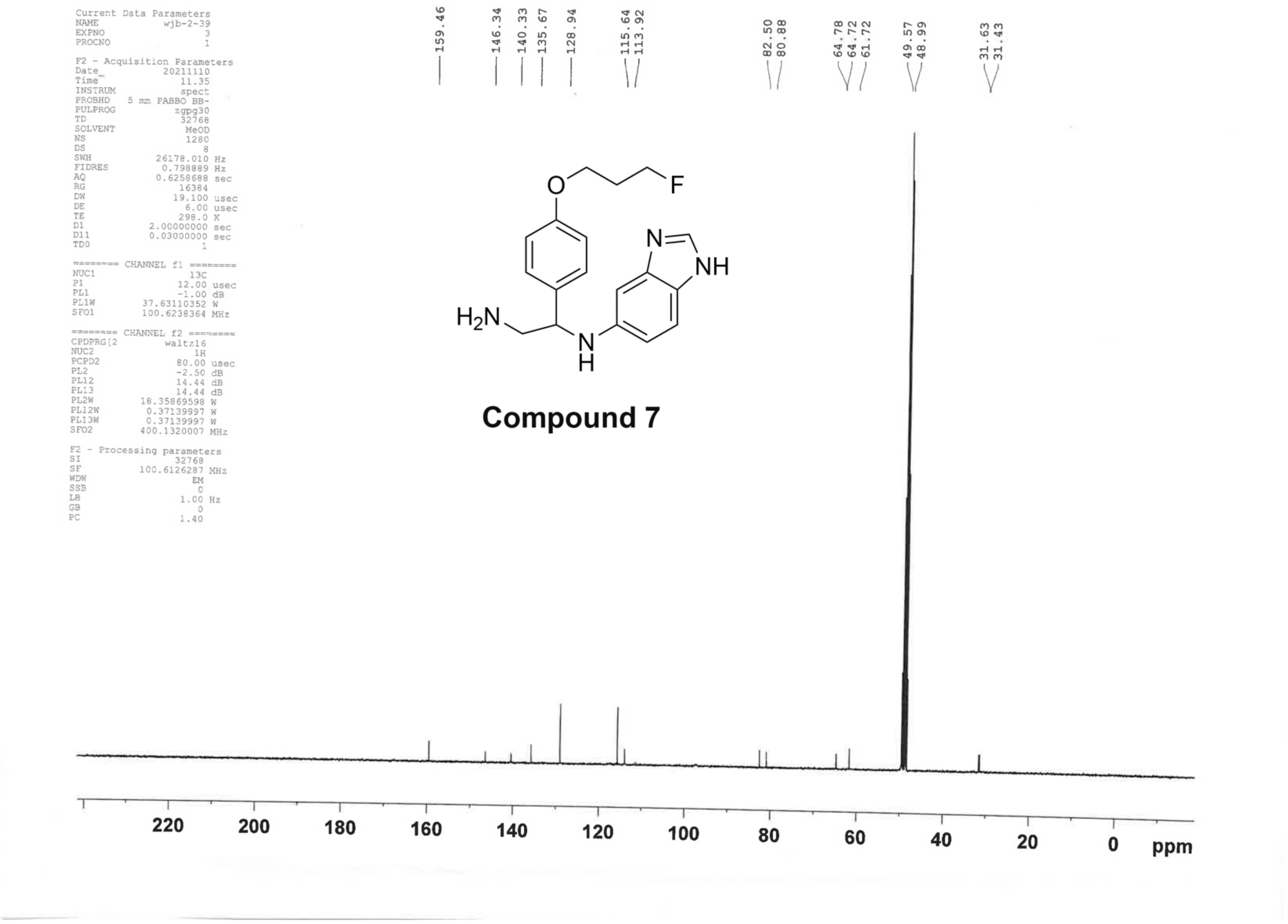

Figure S15. ^{13}C-NMR of compound 7 in methanol-*d*

Synthesis of 1-(1*H*-benzo[*d*]imidazol-5-yl)-5-(4-(3-fluoropropoxy)phenyl)imidazolidin-2-one: Compound 8. To a stirring solution of **7** (101 mg, 0.31 mmol), triethylamine (125 mg, 1.24 mmol) in THF (20 mL) was added 1,1'-Carbonyldiimidazole (100 mg, 0.62 mmol) and stirred at 60°C overnight. The reaction was cooled, diluted with H_2O and EtOAc. The product was extracted 3x with EtOAc. The organic layers were combined, washed with brine, dried over Na_2SO_4, filtered, and concentrated under reduced pressure. The product was purified by flash chromatography (0-100% CH_2Cl_2/(20%MeOH/CH_2Cl_2)) over 18 minutes to afford a final product as a white solid (38 mg, 35% yield). ^{1}H MeOD (600.13 mHz): 9.19 (s, 1H); 7.86 (s, 1H): 7.65 (m, 2H); 7.30 (d, J=8.7 Hz, 2H); 6.86 (d, J=8.8 Hz, 2H); 5.51 (m, 1H); 4.55 (dt, J_1=47.2 Hz, J_2=5.9 Hz, 2H); 4.01 (d, J=6.4 Hz, 2H); 3.97 (t,

J=9.1 Hz, 1H); 3.33 (m, 1H); 2.07 (m, 2H). ^{13}C MeOD (150.9 mHz): 161.9, 160.3, 140.8, 139.5, 133.3, 132.3, 129.0, 128.0, 122.1, 116.0, 115.0, 107.3, 162.9 (d, J=162.9 Hz), 64.8 (d, J=6.0 Hz), 61.8, 48.2, 31.4 (d, J=19.6 Hz).

HRMS (ESI/Q-TOF) m/z: [M + H]+ Calcd for C19H19FN4O2 355.1565; Found 355.1561.

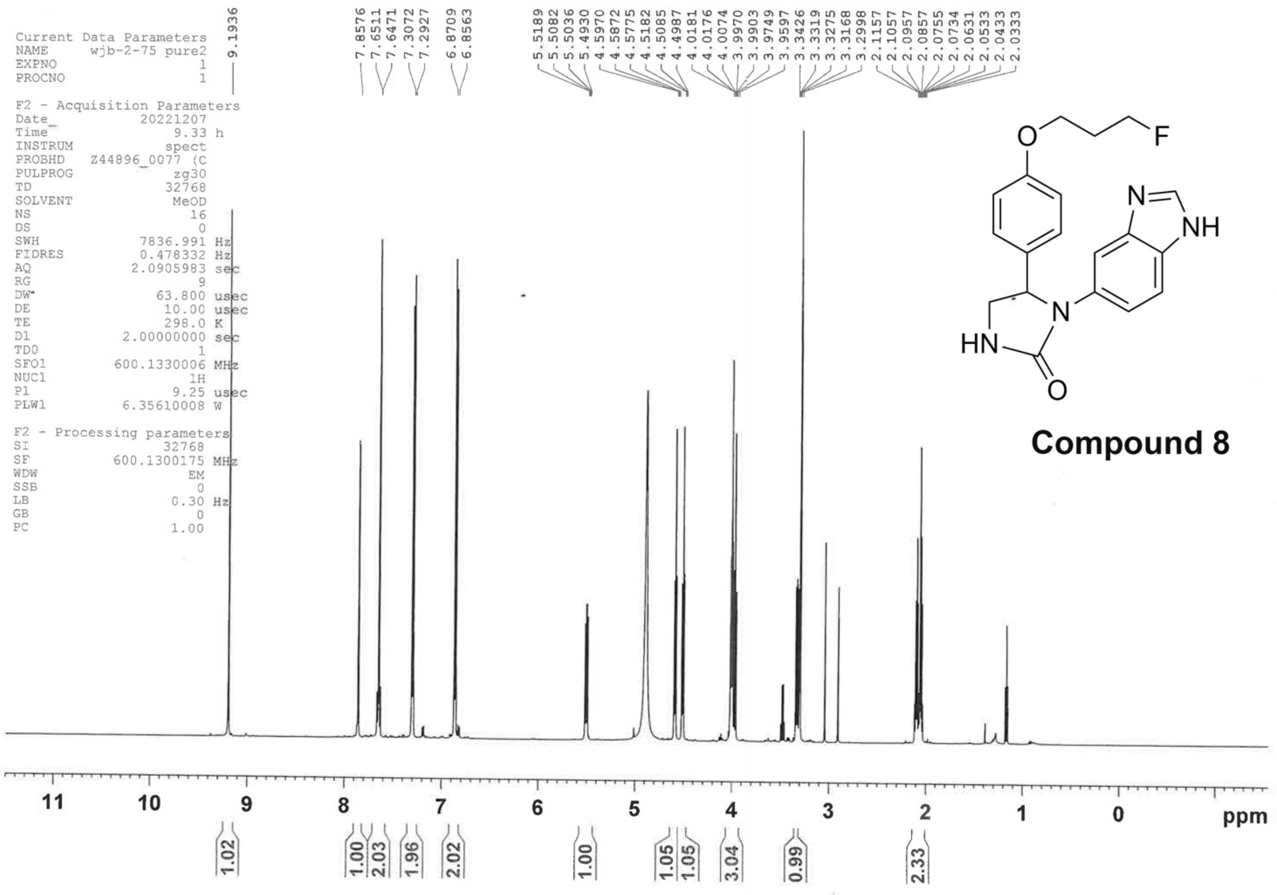

Figure S16. ^{1}H-NMR of compound 8 in methanol-*d*

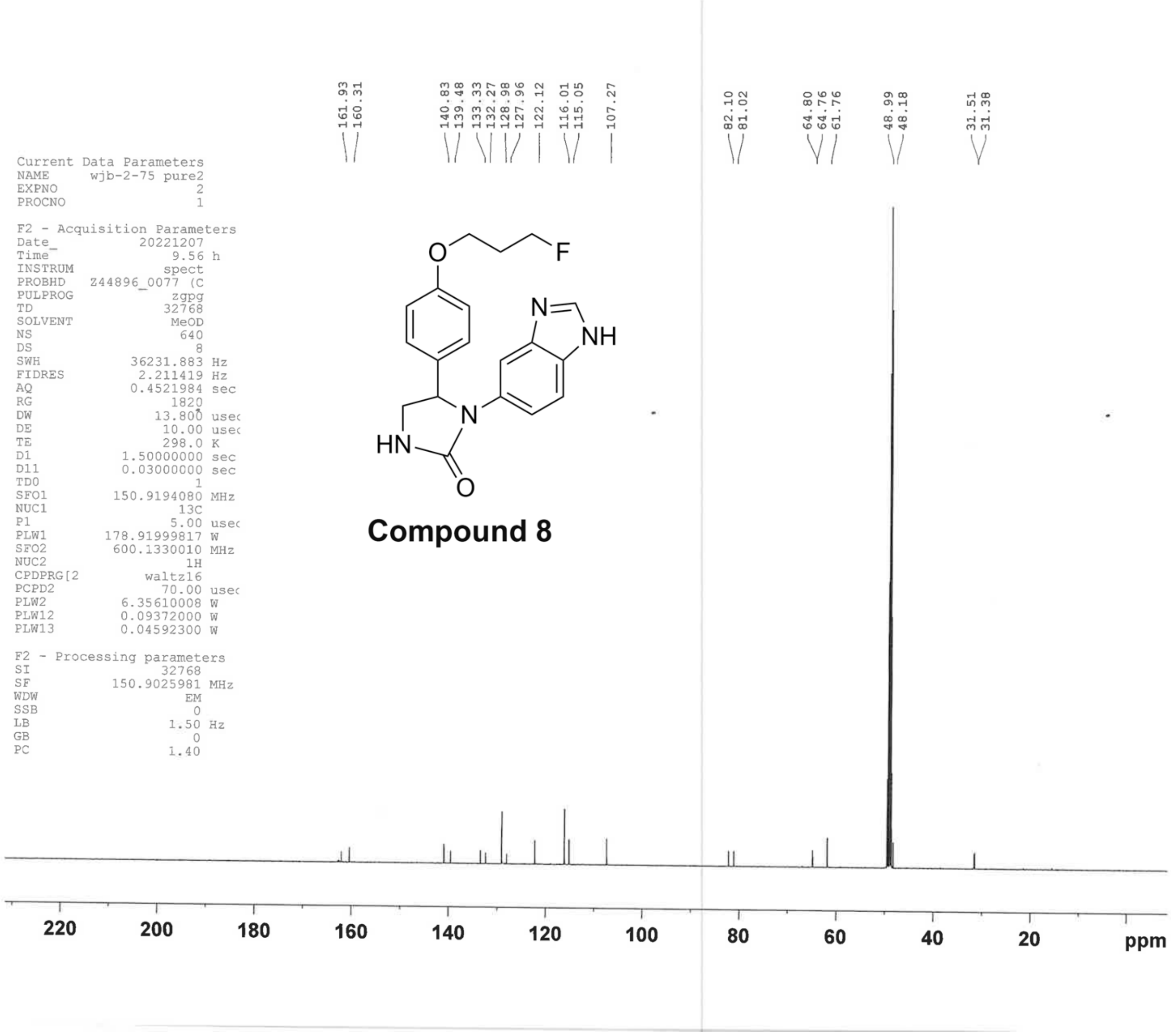

Figure S17. ^{13}C-NMR of compound 8 in methanol-*d*

HPLC of the standard versus [^{18}F]PB0822

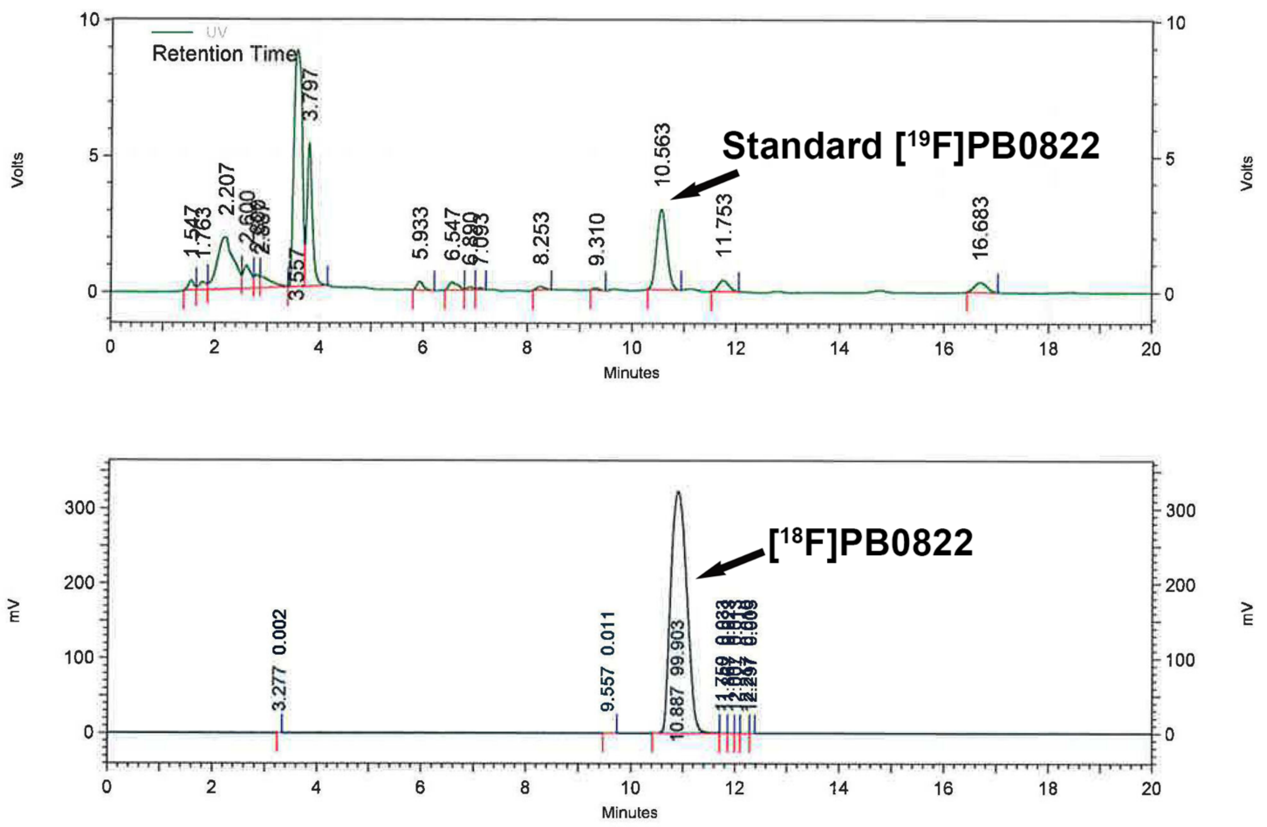

UV Results

Retention Time	Area	Area %	Height	Height %
1.547	10912	1.06	1472	1.60
1.763	11554	1.12	1154	1.26
2.207	164919	15.99	7586	8.27
2.600	34907	3.38	3362	3.66
2.800	12990	1.26	2002	2.18
2.887	24778	2.40	1616	1.76
3.557	378043	36.65	34882	38.01
3.797	152390	14.77	21093	22.98
5.933	10489	1.02	1231	1.34
6.547	13066	1.27	1089	1.19
6.890	3247	0.31	406	0.44
7.093	2101	0.20	298	0.32
8.253	5127	0.50	477	0.52
9.310	2597	0.25	305	0.33
10.563	158310	15.35	11779	12.84

Figure S18. Characterization of the [^{18}F]PB0822 by comparing the retention time in the HPLC with the UV-spectrum of the standard compound [^{19}F]PB0822.

11.753	22430	2.17	1612	1.76
16.683	23682	2.30	1408	1.53
Totals	1031542	100.00	91772	100.00

AID Results

Retention Time	Area	Area %	Height	Height %
3.277	416	0.00	131	0.01
9.557	3133	0.01	312	0.02
10.887	27420986	99.90	1295928	99.69
11.750	8991	0.03	1259	0.10
11.867	6226	0.02	970	0.07
12.007	3587	0.01	682	0.05
12.217	2852	0.01	394	0.03
12.297	1319	0.00	314	0.02
Totals	27447510	100.00	1299990	100.00

Column: Phenomenex Luma C18(2) 00G-4252-E0; 5 um 100A; 250 x 4.6 mm
Mobile Phase: 70% 0.1M AMF 30% AcCN isocratic
Flow Rate: 1 mL/min
UV: 254 nm
Run Time: 20 min

HPLC condition for purification of the [^{18}F]PB0822

Figure S18 (continued). Characterization of the [^{18}F]PB0822 by comparing the retention time in the HPLC with the UV-spectrum of the standard compound [^{19}F]PB0822.

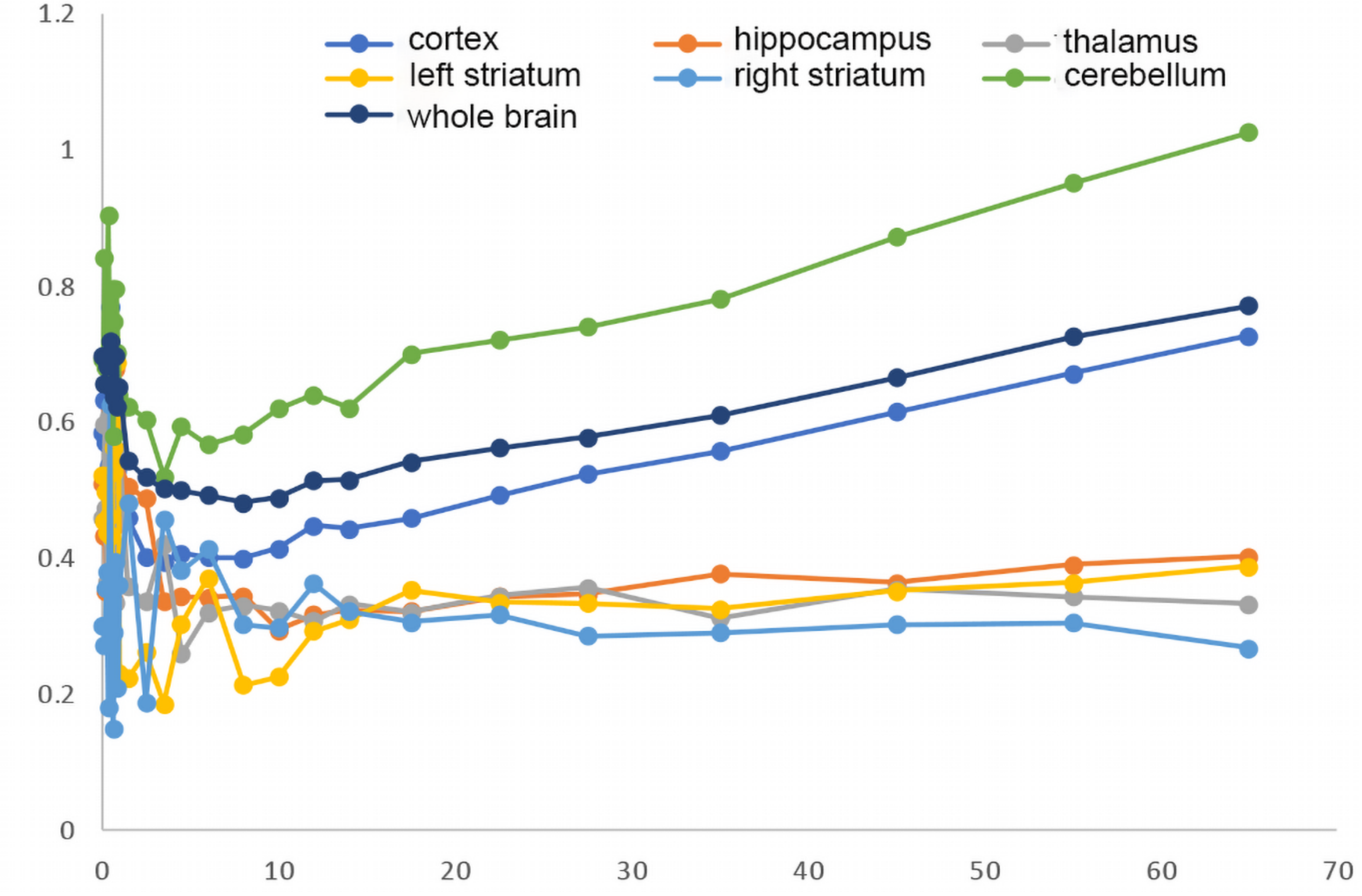

Figure S19. Representative time-activity curve (TAC) data of the dynamic and regional specific uptake of [^{18}F]PB0822 in the brain of a 5XFAD mouse.
